# Feature-Level Reliability of Directional-Kernel Richardson–Lucy Deblurring Under Kernel-Length and Direction Controls

**DOI:** 10.3390/s26165249

**Published:** 2026-08-19

**Authors:** Xiangchen Ku, Runqing Xue, Yichen Liang

**Affiliations:** Department of Mechanical and Electronic Engineering, School of Mechatronics Engineering, Henan University of Science and Technology, Luoyang 471003, China; 240320010153@stu.haust.edu.cn (R.X.); 240320010119@stu.haust.edu.cn (Y.L.)

**Keywords:** motion deblurring, Richardson–Lucy deconvolution, sensitivity analysis, camera-image preprocessing, ORB, SIFT

## Abstract

Image restoration lies between camera acquisition and geometric estimation, but pixel improvements may not transfer to motion estimates. We evaluated directional-kernel Richardson–Lucy (RL) deblurring under kernel-length and direction controls. The restoration analysis covered 3071 paired GoPro, RealBlur-J, and RealBlur-R images. An exploratory feature analysis used a fixed 155-image subset with Oriented FAST and Rotated BRIEF (ORB), scale-invariant feature transform (SIFT), two geometry models, ten random directions, NAFNet, and Restormer. A separate task analysis used ten red–green–blue plus depth (RGB-D) sequences from the Technical University of Munich (TUM) benchmark, synthetic 20 ms exposures, and fixed RGB-D perspective-n-point odometry. Estimated directions contained information relative to random angles, yet the tested global RL branches remained below Blur Input on average. Changes in sequence-mean absolute trajectory error (ATE) RMSE ranged from +0.004 to +0.051 m for ORB and from −0.008 to +0.036 m for SIFT. Seeds were averaged within each sequence before inference. No tested branch achieved a robust ATE improvement across both detectors. Pixel, raw-feature, normalized-feature, geometry-state, and trajectory endpoints produced different method rankings. These findings motivate endpoint-specific evaluation. The task experiment does not validate naturally blurred long-exposure video, a deployed simultaneous localization and mapping system, or sensor hardware.

## 1. Introduction

Camera-image preprocessing is often evaluated through pixel fidelity or visual sharpness, although camera-based measurement ultimately depends on evidence that survives feature extraction, matching, and geometric estimation. Motion blur can reduce this evidence [1,2], while restoration can introduce ringing, gradients, or extra keypoints. A pixel-quality change therefore cannot by itself establish improved sensing reliability.

Camera acquisition produces the image signal. Optional restoration modifies that signal before feature detection and description, and feature correspondences then support geometric fitting. We examine this intermediate link using paired still images.

Directional-kernel Richardson–Lucy (RL) deconvolution is useful here because its main settings can be varied independently [3,4,5,6,7]. These settings include iteration count, point-spread function (PSF) direction and length, boundary handling, and fallback logic. Earlier experiments isolated direction sources at *L* = 13, whereas the length sweep showed that *L* = 5 was substantially less damaging. The feature analysis therefore includes both lengths.

We ask whether simple direction cues outperform random angles and whether a global straight-line PSF improves the blurred input. Restoration questions are evaluated on 3071 pairs, and feature behavior is evaluated on the fixed 155-image subset. A separate ten-sequence Technical University of Munich (TUM) red–green–blue plus depth (RGB-D) experiment tests whether the observed image and feature effects transfer to a ground-truth-referenced camera-motion endpoint under synthetic exposure blur.

The study makes three testable contributions. Within-image random controls and group-aware inference isolate direction-source information from sample composition. Kernel-length, boundary, and ground-truth-assisted (GT-assisted) oracle analyses probe model mismatch within the global straight-line PSF family. Finally, raw and normalized correspondences, geometry states, and sequence-level RGB-D odometry are evaluated together so that restoration preprocessing can be screened against downstream evidence rather than sharpness alone.

## 2. Related Work

Classical blind deconvolution separates latent-image estimation from blur-kernel inference, but both stages remain sensitive to image priors, kernel uncertainty, spatially varying camera motion, and unknown boundaries [5,7,8,9,10,11]. These limitations matter in sensing because a globally plausible restoration can still alter local evidence used for correspondence or measurement. RL and its regularized variants remain useful transparent models for exposing these dependencies [3,4,6,7].

GoPro [12] and RealBlur-J/RealBlur-R [13] provide complementary paired camera data. GoPro derives dynamic-scene blur from high-frame-rate acquisition, whereas RealBlur contains real captured blurred–sharp pairs under two reference-processing settings. Their sequence and scene identifiers also provide natural resampling units for uncertainty estimates, although the grayscale crop used here is not an official benchmark protocol.

Learning-based deblurring has progressed through adversarial, recurrent, multi-stage, convolutional, Transformer, frequency-domain, spatially adaptive, and state-space architectures [14,15,16,17,18,19,20,21,22,23,24,25]. The fixed 155-image tier includes official GoPro-pretrained Restormer [14] and NAFNet [15] checkpoints to determine whether the RL feature pattern persists under modern learned front ends. GoPro is an in-domain control; RealBlur-J and RealBlur-R are cross-dataset sensitivity analyses, not official leaderboard evaluations or separately tuned comparisons.

Recent work has broadened restoration beyond supervised paired-image regression. Self-enhancement and blur-domain conversion address the scarcity and domain mismatch of real paired blur data [26,27], whereas diffusion-based deblurring and collaborative processing introduce alternative training and non-local restoration cues [28,29]. State-space backbones have also emerged as efficient image-restoration architectures [30,31]. More recent studies include an efficient Transformer designed explicitly for deblurring [32], a diffusion-prior framework that separates perceptual restoration from task-oriented utility [33], and a variational K-FISTA formulation with convergence guarantees for deblurring [34]. Related low-level vision studies include detail-enhanced dehazing by Chen, He, and Lu [35] and adaptive mixed-noise suppression by Kusnik and Smolka [36]. These methods address degradations or restoration families beyond the straight-line PSF tested here, but they reinforce the need to examine how preprocessing redistributes image structure. In an adjacent image-analysis setting, a challenge benchmark involving Friedrich and colleagues emphasized dataset coverage and task-defined endpoints [37]. Together, these developments motivate separate reporting of pixel fidelity, correspondence evidence, geometric state, and trajectory error rather than treating visual restoration quality as a sufficient downstream criterion.

Classical motion-blur identification has used spectral zeros, cepstral cues, directional energy, salient structures, and Radon analysis [38,39,40,41,42,43], while the structure tensor estimates dominant local orientation [44]. Camera-image pipelines additionally depend on repeatable feature descriptors and robust geometric fitting; Oriented FAST and Rotated BRIEF (ORB) [45] and random sample consensus (RANSAC) [46] provide a correspondence pipeline, Canny edges [47] provide a complementary contour measure, and scale-invariant feature transform (SIFT) [48] supplies a detector and floating-point descriptor with different contrast and scale behavior.

Feature-oriented deblurring has also been studied with auxiliary sensing and task-level evaluation. Mustaniemi et al. [2] used inertial measurements to estimate spatially varying blur and reported gains in keypoint repeatability, localization accuracy, and 3D reconstruction. MBA-VO [1] modeled the camera trajectory during exposure within visual odometry. The present work has neither inertial measurements nor exposure trajectories: it tests global straight-line PSFs on paired still images and records ORB/scale-invariant feature transform (SIFT) correspondence and pairwise geometry endpoints. Its negative result therefore concerns image-only directional RL, while the NAFNet control tests whether the same endpoint behavior persists after a modern learned front end.

## 3. Materials and Methods

### 3.1. Paired Camera-Image Task and Scope

Given a blurred camera image B and paired sharp reference I, the study evaluates directional-PSF restoration and then tests how paired-image feature measurements depend on kernel length and the specified detector, matcher, and geometric model. The analysis follows the acquired image through restoration preprocessing, feature evidence, and geometric fitting.

(1) B=K∗I+n

**Figure 1:**
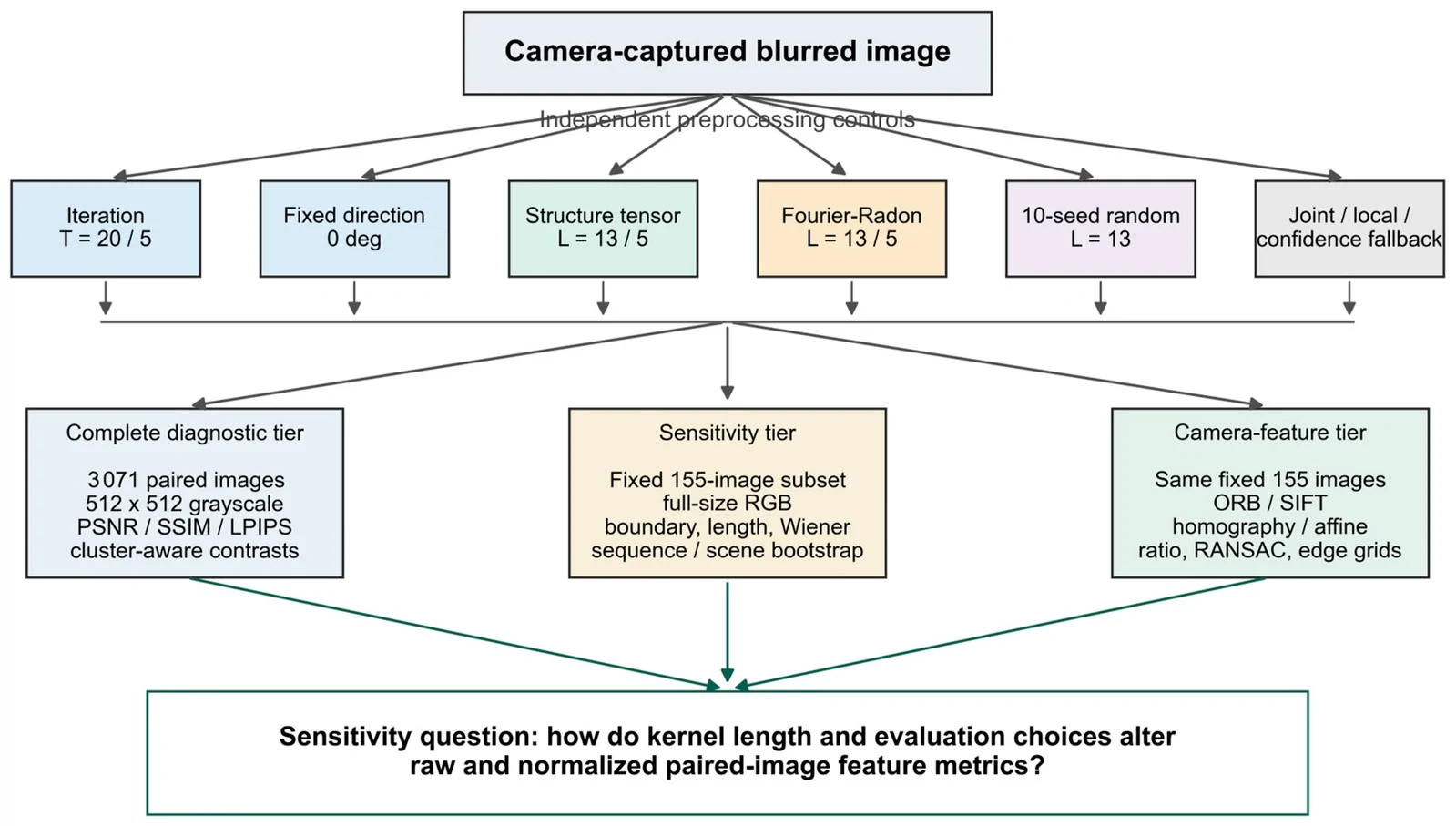
Controlled restoration and feature-sensitivity workflow. Preprocessing branches are parallel controls; the feature tier crosses *L* = 13 and *L* = 5 with ORB/SIFT, homography/affine fitting, and matching-parameter sensitivity. ORB, Oriented FAST and Rotated BRIEF; SIFT, scale-invariant feature transform; PSNR, peak signal-to-noise ratio; SSIM, structural similarity index; LPIPS, Learned Perceptual Image Patch Similarity; RANSAC, random sample consensus.

In Equation (1), *B* denotes the blurred image, *I* denotes the latent or reference image, *K* denotes the tested blur kernel, *n* denotes residual noise and model mismatch, and ∗ denotes two-dimensional convolution. The paired reference was used only for evaluation or for explicitly labeled oracle-angle diagnostics. Figure 1 summarizes the independent experimental controls.

### 3.2. Richardson–Lucy Update and Kernel Rasterization

All RL branches used the multiplicative update in Equation (2), following the classical Richardson–Lucy formulation [34], with non-negative image intensities normalized to [0, 1].

(2) $$I^{\left(t+1\right)}=I^{\left(t\right)}⊙\left[K^{T}*\frac{B}{K*I^{\left(t\right)}+\varepsilon_{RL}}\right],\;\varepsilon_{RL}=10^{-12}$$

Here, *K*^T^ denotes the flipped kernel, ⊙ denotes element-wise multiplication, division is element-wise, and *ε* = 10^−12^. Outputs were clipped to [0, 1]. The 21 × 21 line PSF was rasterized by rotating a centered horizontal segment and normalizing it to unit sum.

### 3.3. Direction-Source Controls


(3) $$\Delta_{\theta }=\left|\hat{\theta }-\theta \right|,e_{axial}=min\left(\Delta_{\theta },\;\;180^{\circ }-\Delta_{\theta }\right)$$

The primary direction comparison fixed the kernel length at 13 pixels, and the RL iteration count at *T* = 5. Fixed RL used 0°. The structure-tensor angle-only branch used the dominant orientation returned by a Sobel gradient structure tensor [44] but discarded its heuristic length. This design ensured that comparisons with the random-angle and Fourier–Radon branches isolated the direction source while keeping kernel length and iteration count fixed. Gradient energy, the 60th-percentile support mask, and the normalized second-moment matrix are defined in Equation (4)–(6). The dominant tensor orientation and its mapping to the line-PSF direction are given in Equations (7) and (8), respectively.

(4) $$g\left(x,y\right)=\sqrt{B_{x}^{2}\left(x,y\right)+B_{y}^{2}\left(x,y\right)}$$

(5) $$M=\left\{\left(x,y\right):g\left(x,y\right)>Q_{0.60}\left(g\right)\right\}$$

(6) $$J=\frac{1}{\left|M\right|}\sum_{\left(x,y\right)\in M}\left[\begin{array}{cc} B_{x}^{2}\left(x,y\right) & B_{x}\left(x,y\right)B_{y}\left(x,y\right)\\ B_{x}\left(x,y\right)B_{y}\left(x,y\right) & B_{y}^{2}\left(x,y\right) \end{array}\right]=\left[\begin{array}{cc} J_{xx} & J_{xy}\\ J_{xy} & J_{yy} \end{array}\right]$$

(7) $$\theta_{g}=\frac{1}{2}atan2\left(2J_{xy},J_{xx}-J_{yy}+\varepsilon_{J}\right)\frac{180{}^{\circ}}{\pi },\;\varepsilon_{J}=10^{-12}$$

(8) $$\theta_{ST}={wrap}_{\left[-90{}^{\circ},\left.90{}^{\circ}\right)\right.}\left(\theta_{g}-90{}^{\circ}\right)$$

The Fourier–Radon branch followed classical spectral and Radon approaches to linear motion-blur identification [38,41]. The direction-estimation image was resized to a maximum side length of 256 pixels. We then applied a two-dimensional Hann window, formed the centered log power spectrum, and suppressed an 8-pixel central disk.

The selected direction was the 1° Radon projection with maximum variance. Angles were treated as axial directions in [−90°, 90°) and passed directly to the OpenCV line-PSF rotation convention. Equation (3) defines axial angular error modulo 180°. Equations (9) and (10) describe the spectrum processing, and Equations (11) and (12) define angle selection and conversion.

The 168-condition calibration used kernel lengths of 7, 13, and 19 pixels, noise standard deviations of 0 and 0.01, and four texture classes. The textures were GoPro, RealBlur-J, and RealBlur-R reference crops plus a procedural multiscale texture. Synthetic convolution used reflection padding. This experiment checked only the coordinate convention; it neither validates direction ground truth in real motion blur nor tunes the estimator or RL parameters. The conditions share textures and parameter levels and are therefore correlated rather than independent replications. Table 1 gives a compact summary, and Supplementary Table S6 contains condition-level results.

(9) $$P\left(u,v\right)=\ln\left[1+{\left|F_{c}\left\{w\left(x,y\right)\left[B_{s}(x,y)-\bar{B_{s}}\right]\right\}\right|}^{2}\right]$$

(10) $$\tilde{P}\left(u,v\right)=\left\{\begin{array}{cc} median\left(P\right) & {\left(u-u_{0}\right)}^{2}+{\left(v-v_{0}\right)}^{2}<r_{c}^{2}\\ P\left(u,v\right) & otherwise \end{array}\right.,r_{c}=8$$

(11) $$\Phi =\left\{0^{\circ },1^{\circ },\ldots ,179^{\circ }\right\},\;\phi^{⋆}=\arg\underset{\phi \in \Phi }{max}\;{Var}_{\rho }\left[R\left\{\tilde{P}\right\}\left(\rho ,\phi \right)\right]$$

(12) $$\theta_{FR}={wrap}_{\left[-90{}^{\circ},\left.90{}^{\circ}\right)\right.}\left(\phi^{⋆}\right)$$

Random-angle RL used 10 fixed seeds (20260701–20260710), with each seed–image pair mapped deterministically to [−$90^{{}^{\circ}}$, $90^{{}^{\circ}}$) using SHA-256. The GT-assisted joint oracle used the fixed 155-image manifest, grayscale 512 × 512 center crops, *T* = 5, reflection padding, L ∈ {5, 9, 13, 17, 21}, and every integer angle from −90° to 89°. The paired reference selected the maximum-PSNR (θ*, L*) for each image. This 900-candidate search is a selection-optimistic upper bound and not a deployable estimator; angle-only L = 13 and estimated branches were recomputed under the same implementation.

The synthetic calibration results are summarized in Table 1. The direct Fourier–Radon mapping achieved substantially lower axial angular error than the 90°-rotated mapping and the structure-tensor estimator.

### 3.4. Adaptive, Local, Confidence, and Wiener Branches

The original adaptive branch jointly used the structure-tensor angle and a heuristic length derived from anisotropy and Laplacian variance. Its confidence score combined gradient anisotropy with a sharpness term. When *c* < 0.08, the fallback branch returned “Blur Input”. The inherited threshold *c* = 0.08 was retained solely as a diagnostic operating point. It was neither optimized nor independently validated and is not proposed as a deployment threshold. A 2 × 2 local branch estimated the same heuristic in overlapping tiles. Equations (13)–(17) define the anisotropy score, sharpness term, length heuristic, and fallback rule.

(13) $$a=\frac{\sqrt{{\left(J_{xx}-J_{yy}\right)}^{2}+4J_{xy}^{2}}}{J_{xx}+J_{yy}+\varepsilon_{J}}$$

(14) $$v_{L}=Var\left[\nabla^{2}B\right]$$

(15) $$\tilde{L}=clip\left[round\left(5+16a+\frac{4}{v_{L}+0.2}\right),3,S\right],\;L=\tilde{L}+1\left(\tilde{L}\;is\;even\right)$$

(16) $$c=clip\left[amin\left(1,\frac{v_{L}}{0.01}\right),0,1\right]$$

(17) $$I_{gate}=\left\{\begin{array}{ll} I_{adapt}, & c\geq \tau_{c},\\ B, & c<\tau_{c}, \end{array}\right.\tau_{c}=0.08$$

The all-pair table retains a nominal, untuned Wiener branch with a balance = 0.015 only for continuity with the earlier analysis. It is not presented as an optimized or representative Wiener baseline. A separate sensitivity control evaluates balances 0.001, 0.005, 0.015, 0.05, and 0.1 on the fixed 155-image subset, without selecting a post hoc winner or making a method-family claim.

### 3.5. Public Data and Preprocessing

The all-pair analysis covered 1111 GoPro, 980 RealBlur-J, and 980 RealBlur-R pairs [12,13]. Images were decoded as grayscale, cropped to common overlapping dimensions, and center-cropped to 512 × 512 pixels. Restoration was assessed with peak signal-to-noise ratio (PSNR), the structural similarity index (SSIM) [49] and Learned Perceptual Image Patch Similarity with an AlexNet backbone (LPIPS-Alex) [50]. For LPIPS-Alex, grayscale luminance was replicated across three channels.

The 155-image subset was generated before the added analyses from seed 20260710. Within each dataset group sorted by image ID, a group-specific generator was initialized from the first eight bytes, interpreted as little-endian, of SHA-256 (“20260710:dataset:group_id”). Five GoPro frames were sampled without replacement from each of 11 sequences, and one pair was sampled from each of 50 scenes in RealBlur-J and RealBlur-R; sampled indices were sorted. No result-dependent image exclusion was applied. The complete IDs, group labels, paths, seed, and selection rule are retained in Supplementary Data and Code S1.

Subset comparability was assessed descriptively before feature interpretation. We compared dataset composition, group coverage, and Blur Input PSNR and SSIM between the fixed subset and all available pairs. We also summarized baseline ORB and SIFT $q_{norm}$ and geometry success under the declared homography setting.

The subset selection was independent of experimental results and covered every sequence or scene group, ensuring basic coverage. These checks support coverage; they do not establish independent representativeness or an adequately powered confirmatory validation sample. The feature analyses were post hoc extensions, and the cluster sizes were fixed at 50 scenes per RealBlur dataset and 11 GoPro sequences; therefore, no retrospective confirmatory statistical power assessment was conducted. The confidence interval widths at the cluster level reflect the attainable sampling precision at this exploratory tier.

### 3.6. Statistical Protocol

Paired PSNR changes were summarized using the mean, a cluster-bootstrap 95% percentile confidence interval (CI), the median, the interquartile range (IQR), the improved-image proportion, and the improved-group proportion. Cluster resampling followed the nonparametric cluster-bootstrap principle [51]. Bootstrap resampling used 10,000 replicates and seed 20260711. GoPro sequences and RealBlur scenes were sampled with replacement while retaining all images from each sampled group. For a per-image change or direct contrast d_i, a sequence or scene was classified as improved (or as favoring the first method) when the arithmetic mean of d_i within that group was greater than zero. The improved-group or better-group proportion was the number of such positive-mean groups divided by the total number of groups.

Seed-level means and standard deviations were retained as descriptors of algorithmic sensitivity. All contrasts were first computed per image and then aggregated to the sequence or scene cluster level. For the two primary GoPro restoration contrasts and the primary *L* = 5 feature contrasts, we subjected the 11 sequence-level mean effects to an exact two-sided Rademacher randomization test by enumerating all $2^{11}$ sign assignments. The *p*-value was the proportion of sign-flipped absolute mean effects at least as large as the observed absolute sequence-mean effect. Frames were not treated as independent randomization units.

A positive confidence outcome was defined based on the ungated joint angle–length output relative to the Blur Input: for fidelity, the positive label was ΔPSNR > 0; for perceptual similarity, the positive label was ΔLPIPS < 0. We report dataset-specific, macro-average, and pooled receiver operating characteristic area under the curve (ROC-AUC), average precision (AP), and positive prevalence [52,53].

Confidence intervals were computed from 5000 sequence- or scene-cluster bootstrap replicates (seed 20260711). Replicates lacking either outcome class were excluded, and valid and excluded counts were recorded. For the ungated joint branch, the feature-subset label was Δ$q_{norm}$ > 0, and its ROC-AUC and AP intervals were based on 10,000 group-bootstrap replicates. Threshold curves show the acceptance rate, mean gated change, false accepts, and false rejects; these curves are presented for diagnostic illustration only and were not used to select c.

### 3.7. Boundary Sensitivity

The full-size sensitivity analysis recomputed the fixed, joint adaptive, structure-tensor angle-only, Fourier–Radon, confidence-fallback, and 10-seed random-angle branches at *T* = 5 under explicit zero and reflection convolution boundaries. This control was included because unknown or imposed image boundaries can materially affect deconvolution [5]. For the 21 × 21 PSF, valid-interior metrics excluded a 10-pixel border. Full-image PSNR, valid-interior PSNR, and border root mean square error (RMSE) were paired with identically defined blurred-input metrics. Exploratory border-only regional values are confined to Supplementary Table S5.

### 3.8. Fixed Experimental Settings

Table 2 summarizes the experimental settings and records the provenance and analytical role of *T*, *L*, *c*, *λ*, and *τ*.

### 3.9. Kernel-Length and Wiener Sensitivity

A finite kernel-length sensitivity used the fixed 155-image manifest and the grayscale 512 × 512 crop. At *T* = 5 and with reflection padding, we evaluated *L* ∈ {5, 9, 13, 17, 21} for the fixed, structure-tensor, and Fourier–Radon direction sources. Each dataset–direction–length combination was summarized by mean ΔPSNR, median, IQR, and sequence- or scene-cluster bootstrap 95% CI (10,000 replicates; seed 20260712). The Wiener balance sweep used the same images, crop, fixed 0° *L* = 13 PSF, and balances {0.001,0.005,0.015,0.05,0.1}; its fast Fourier transform (FFT) implementation has the circular-boundary convention of skimage.restoration.wiener.

### 3.10. Full-Size RGB Sensitivity

The same 155 pairs were evaluated at full resolution in RGB for Blur Input, fixed *T* = 5, structure-tensor angle-only, Fourier–Radon, and confidence fallback. Direction and confidence were estimated from grayscale luminance and the resulting PSF was applied independently to each RGB channel with reflection padding. PSNR used data_range = 1.0. SSIM used data_range = 1.0, win_size = 7, and channel_axis = 2. This sensitivity analysis is not an official GoPro or RealBlur leaderboard protocol.

### 3.11. Camera-Image Feature Sensitivity

The feature extensions used the fixed 155-image full-size subset. They covered *L* = 5, SIFT, affine fitting, parameter grids, random angles, failure transitions, ORB caps, and learned models. These post hoc sensitivity analyses were not preregistered. Fixed 0°, structure-tensor, Fourier–Radon, and random-angle RL used *T* = 5, reflection padding, and *L* = 5. Deterministic *L* = 13 branches were retained for the length comparison. Random angles used seeds 20260701–20260710 and the same SHA-256 image mapping as the all-pair analysis. Seed outputs were averaged within each image before sequence- or scene-cluster bootstrap inference.

(18) $$q_{norm}=\left\{\begin{array}{cc} \frac{N_{inlier}}{min\left(N_{kp,candidate},N_{kp,reference}\right)} & min\left(N_{kp,candidate},N_{kp,reference}\right)>0\\ 0 & otherwise \end{array},\right.$$

The primary feature endpoint was the normalized ORB-homography RANSAC inlier count at ratio 0.75 and a 3-pixel threshold. Equation (18) divides raw inliers by the smaller candidate/reference keypoint count. This count bounds one-to-one correspondences and provides a symmetric, bounded opportunity normalization. Reference-only and candidate-only denominators are asymmetric. Good matches depend directly on the ratio threshold, and the geometric mean does not bound correspondence count. However, restoration can add candidate keypoints and lower the normalized endpoint. We therefore interpreted $q_{norm}$ with raw inliers, candidate keypoints, good matches, inlier ratio, and geometry success. When either candidate or reference keypoints were absent, the zero-denominator rule set $q_{norm}=0$.

ORB used nfeatures = 2000 for the declared primary analysis. A post hoc cap analysis evaluated nfeatures = 1000, 2000, 4000, 8000, 12,000, 16,000, 20,000, and 24,000. It covered Blur Input and deterministic L = 5 branches while holding matching and geometry settings fixed. From 8000 onward, the limit increased by 4000 until one of two stopping conditions was met. First, reference and candidate cap proportions had to be at most 5% in every dataset–method cell. Alternatively, consecutive limits had to change mean paired Δq by at most 0.005 without reversing its sign in any cell. The first qualifying limit was retained, and all intermediate limits are reported.

The primary feature analysis retained all 155 images and assigned zero inliers to descriptor or geometry failures. A sensitivity analysis retained only images for which Blur Input succeeded under the same detector and geometry model. We recorded all four baseline-to-processed geometry transitions.

Detector, geometry, matching, cap, confidence, and learned-model comparisons were secondary or exploratory. The 18 parameter-grid configurations share the same images and overlapping settings; they are correlated sensitivity conditions, not independent replications. Their summaries emphasize effect ranges and confidence intervals rather than vote counts.

Before applying the multiple-comparison correction, we defined a secondary family of 12 comparisons spanning three datasets, two detectors, and two estimated L = 5 directions. Each direction was compared against the within-image mean of 10 random seeds, using a homography model, a ratio threshold of 0.75, and a 3-pixel RANSAC threshold; tests were based on sequence- or scene-mean effects. For the 11 GoPro sequences, exact tests were performed by enumerating all sign assignments; for each 50-scene RealBlur dataset, 200,000 Monte Carlo sign permutations were used (seed 20260807). The resulting 12 *p*-values were uniformly adjusted using the Benjamini–Hochberg false discovery rate (BH-FDR). This family of tests was defined post hoc and was not prospectively preregistered. The larger parameter grids remain exploratory, and no hypothesis-by-hypothesis significance claims are made.

### 3.12. Implementation Details

The experiments were conducted under Microsoft Windows 10 (Version 22H2, build 19045) on a custom desktop workstation based on an MS-7D90 motherboard (Micro-Star International Co., Ltd., New Taipei City, Taiwan). The workstation was equipped with a 13th Gen Intel Core i5-13400F processor (Intel Corporation, Santa Clara, CA, USA) and an AMD Radeon RX 6750 GRE 12GB graphics processor (Advanced Micro Devices, Inc., Santa Clara, CA, USA). The principal numerical analyses and PyTorch inference were CPU-based and did not use CUDA. The NAFNet ONNX stream in the task-level experiment was executed through DirectML on the AMD graphics processor.

The software environment comprised Python (Version 3.12.7), NumPy (Version 1.26.4), SciPy (Version 1.13.1), OpenCV-Python (Version 4.10.0.84), scikit-image (Version 0.24.0), scikit-learn (Version 1.5.1), pandas (Version 2.2.2), Matplotlib (Version 3.9.2), seaborn (Version 0.13.2), Pillow (Version 10.4.0), LPIPS (Version 0.1.4), PyTorch (Version 2.11.0+cpu), ONNX Runtime DirectML (Version 1.20.1), and pytest (Version 7.4.4).

OpenCV-Python provided image decoding, convolution, ORB, SIFT, Canny, RANSAC, homography, affine-transform, and perspective-n-point implementations. Scikit-image provided the Radon-transform and Wiener-deconvolution implementations. Scikit-learn was used to calculate ROC-AUC and average precision. Matplotlib and seaborn were used for statistical visualization, whereas pytest was used for integrity and reproducibility testing.

Images were decoded as uint8 arrays, converted to float32 values in [0, 1], and clipped to this range after restoration. Reflection-boundary convolution used cv2.BORDER_REFLECT, and the adjoint RL update used the spatially flipped point-spread function. Complete environment metadata, dependency versions, random-number seeds, manifests, integrity tests, and rerun commands are provided in Supplementary Data and Code S1.

All image, depth, and trajectory inputs were obtained from the cited public GoPro, RealBlur, and TUM RGB-D datasets. The authors did not operate physical cameras, laboratory instruments, custom sensing devices, or use chemicals or reagents in this computational study.

The fixed quantities served specific factor-isolation roles. *T* = 5 was the low-iteration control, *L* = 13 isolated direction-source effects, and *L* = 5 was the lower-disturbance sensitivity control. The inherited *c* = 0.08 value was a historical diagnostic operating point. *T*, *L*, and c were not treated as optimal or independently calibrated parameters. Only λ and τ for the newly added RL-TV and discrepancy-stopping branches were selected by group-disjoint internal calibration and then locked for validation. Table 2 records these distinctions.

### 3.13. Modern Learned-Restoration Controls

The official NAFNet implementation was obtained from the Megvii Research repository and evaluated at Git commit 2b4af71ebe098a92a75910c233a3965a3e93ede4. The official Restormer implementation was obtained from its authors’ repository and evaluated at Git commit 68dc6ac472db26f16361150cb7a96a1bc87da93f. Both repositories retained their original Git metadata, and the evaluated working trees contained no local code modifications.

The official GoPro-pretrained NAFNet-GoPro-width64 checkpoint had the SHA-256 identifier 329D3AB4077B8D6B7FF61DE376E483714667960BF85BE027BF4335CDA701196F. The official GoPro-pretrained Restormer motion-deblurring checkpoint had the SHA-256 identifier 194E38FB5B607C9DC5A5B3E08E65B2E79EE2BF0EF5048E0612F6B2FF2F79DA31.

Official GoPro-pretrained NAFNet [15] and Restormer [14] checkpoints were evaluated without retraining or dataset-specific tuning. Full-size RGB inputs were padded only to each architecture factor, restored, cropped to original size, and clipped to [0, 1]. Pixel metrics used RGB output and luminance. ORB/SIFT evaluation used the same grayscale conversion, ratio 0.75, homography/affine fitting, and 3-pixel RANSAC threshold as the classical branches. Differences from Blur Input and deterministic *L* = 5 RL outputs were formed within each image before sequence/scene cluster bootstrap.

### 3.14. Calibrated Discrepancy-Stopping and RL-TV Controls

Two post hoc, protocol-fixed control branches used the Fourier–Radon direction, *L* = 5, a 21 × 21 PSF, and reflection padding. Groups were ordered by a stable SHA-256 key before any branch was run. The calibration split comprised 3 of 11 GoPro sequences and 10 of 50 scenes from each RealBlur dataset (35 images), while the validation split consisted of the remaining 8 sequences and 40 scenes from each RealBlur dataset (120 images). As these groupings had appeared in prior analyses, the split provides internal—rather than untouched external—validation for the new branches.

The first branch employed unregularized RL with discrepancy-based stopping. The second branch adopted the multiplicative TV-regularized update of Dey et al. [7]. Equation (19) defines this update:

(19) $$I^{t+1}=I^{t}⊙\frac{K^{T}*\frac{B}{K*I^{t}+\varepsilon }}{1+{\lambda g}_{TV}\left(I^{t}\right)}$$

The global regularization weight λ was chosen from {10^−4^, 3 × 10^−4^, 10^−3^, 3 × 10^−3^, 10^−2^}, and the global discrepancy multiplier from {0.5, 1, 2, 4}. Selection maximized the macro, group-balanced calibration change in the primary ORB-homography $q_{\mathrm{norm}}$ endpoint, with ΔPSNR and smaller parameter values serving only as tie-breakers. Both parameters were then locked for validation.

A noise scale was estimated from each blurred image and converted to an effective count scale. At each iteration, the image-domain Poisson deviance between B and $\;K*I^{t}\;$ was computed. Equation (20) defines this deviance:

(20) $$D_{P}\left(B\left\|K*I^{t}\right.\right)=\frac{2}{N}\sum_{p}\left[y_{p}\ln\frac{y_{p}}{\mu_{p}}-y_{p}+\mu_{p}\right]$$

where $y_{p}$ and $\mu_{p}$ denote the effective observed and predicted counts at pixel p, respectively.

Iterations stopped at the first t≥2 where the per-pixel deviance did not exceed the locked multiplier; T=30 served as a safety upper bound. Since raw photon counts and calibrated sensor-noise measurements were unavailable, this rule constitutes a calibrated, quasi-Poisson image-domain discrepancy criterion rather than a physically grounded camera-noise theorem.

### 3.15. Ground-Truth-Referenced RGB-D Odometry Experiment

We used ten frozen sequences from the public TUM RGB-D benchmark [54]. The technical synchronization required RGB–depth pairs to be within 0.02 s, and the ground-truth trajectory had to cover the entire synthetic exposure interval; both rules were applied before any method outputs were generated. The registered 640 × 480 RGB-D frames used a focal length of 525 pixels and a principal point of (319.5, 239.5), with a depth scale of 5000 units per meter. No method-specific exclusion of frames or sequences was permitted.

Blur Input was rendered as a 20 ms uniform global exposure with nine temporal samples. Ground-truth translations were obtained by linear interpolation, and rotations used quaternion spherical interpolation. The central RGB-D surface was forward-projected to each sub-pose with a z-buffer. Uncovered pixels retained the central RGB value, and no photometric noise was added. Ground truth was used only to synthesize the exposure and score the trajectories; restoration and odometry did not receive the target pose. This experiment evaluates trajectory-conditioned synthetic exposure on real RGB-D sequences with measured camera trajectories. It does not constitute validation on naturally blurred long-exposure video or a deployed simultaneous localization and mapping (SLAM) system.

Every sequence retained identical resolution, intrinsic calibration, timestamps, frame count, and ordering across all inputs. The RL inputs included fixed, structure-tensor, and Fourier–Radon branches at L=5, as well as fixed and structure-tensor RL at L=13, confidence fallback, and ten random-angle seeds at  L=5. We also included the official GoPro-pretrained NAFNet and the official motion-deblurring Restormer. RL used T=5 and reflection padding throughout. A preprocessing exception returned Blur Input instead of dropping a frame when conditions were not met. An integrity gate required zero such fallbacks for both learned-model streams.

The downstream pipeline estimated frame-to-frame motion from depth-supported correspondences. ORB with $n_{\mathrm{features}}=2000$ served as the primary detector, with SIFT as a secondary option; both used a 0.75 ratio test. Efficient perspective-n-point (EPnP) random sample consensus employed a 3-pixel threshold, 100 iterations, and 0.999 confidence, followed by iterative refinement after the RANSAC estimate.

Frames with a failed estimate were retained, an identity relative transform was applied, and the failure count was incremented. The primary endpoint was absolute trajectory error (ATE) RMSE after rigid special Euclidean group SE(3) alignment without scale. Secondary endpoints included median ATE, translational and rotational relative pose error (RPE), perspective-n-point (PnP) success rate, number of initialization failures, trajectory coverage, number of matches, inliers, inlier ratio, and odometry runtime per frame pair.

Runtime covers only the downstream odometry. Preprocessing time is not comparable between CPU and DirectML and is therefore not used for efficiency claims. The RGB-D PnP pipeline has no relocalization module, so relocalization counts are undefined.

The inferential unit was the sequence. For each detector and method, the paired effect is defined by Equation (21):

(21) $$d_{j}={ATE}_{RMSE}\left(Processed,j\right)-{ATE}_{RMSE}\left(BlurInput,j\right)$$

The ten random-angle seeds were averaged within each sequence before inference. We report the sequence mean, median, proportion of improved sequences, and leave-one-sequence-out range. Uncertainty analyses used 20,000 sequence bootstrap replicates and wild-cluster intervals, and we also report the exact paired sign test and exact sign-flip test. Because only ten sequences were available, these analyses constitute task-specific sensitivity estimates. The frozen protocol and its SHA-256 digest were fixed before the multi-sequence outcomes were generated. The overall analysis hierarchy, inferential units, and multiplicity treatment across all experimental tiers are summarized in Table 3.

### 3.16. Post Hoc Distortion and Precision Analysis

On the fixed 155-image subset, we quantified three ground-truth-referenced distortion measures for Blur Input and the fixed-direction, structure-tensor, and Fourier–Radon branches at L=5 and L=13. The analysis was performed in grayscale within the valid interior region obtained by excluding a 10-pixel border. Reference edge pixels were defined as those where the Sobel gradient magnitude in the reference image exceeded the 85th percentile, and were dilated by 2 pixels. The edge-envelope violation measured the average amount by which the candidate exceeded the 5×5 local reference intensity envelope within this edge band. Gradient-magnitude distortion was defined as the mean absolute difference between the candidate and reference Sobel magnitudes, divided by the mean reference gradient magnitude. The edge-band Laplacian residual energy was the mean absolute Laplacian difference divided by the reference Laplacian energy. All metrics were expressed as paired changes from Blur Input, with positive values indicating greater distortion.

For the L=5 branches, Spearman rank correlations were used to relate each distortion change to changes in the number of candidate keypoints, raw RANSAC inliers, and normalized inliers. Confidence intervals were based on 10 000 sequence- or scene-cluster bootstrap replicates (seed 20260808). When a variable was constant—as occurred for candidate-keypoint changes in GoPro under the 2000-feature cap—the correlation was regarded as not estimable. These analyses test mechanistic associations rather than causal relationships.

We also evaluated the precision of the fixed-subset feature contrasts at the sequence or scene level. For each contrast, image-level effects were averaged within groups. We report the group-mean effect, a two-sided t-based 95% confidence interval and its half-width, and the minimum detectable effect (MDE) for α=0.05 and 80% power, computed from the noncentral t distribution using the observed between-group standard deviation. The MDE reflects the effect size detectable by this study and does not serve as an equivalence margin or as evidence that smaller effects are absent.

## 4. Results

Results are organized by evidence scale. Section 4.1, Section 4.2 and Section 4.3 cover restoration and confidence across 3071 grayscale pairs. Section 4.4, Section 4.5, Section 4.6, Section 4.7, Section 4.8 and Section 4.9 examine the fixed 155-image subset and group-held-out controls. Section 4.10 reports the separate ten-sequence RGB-D odometry experiment. Section 4.11 reports the post hoc distortion and precision analyses.

### 4.1. All-Pair Performance Distributions

**Figure 2:**
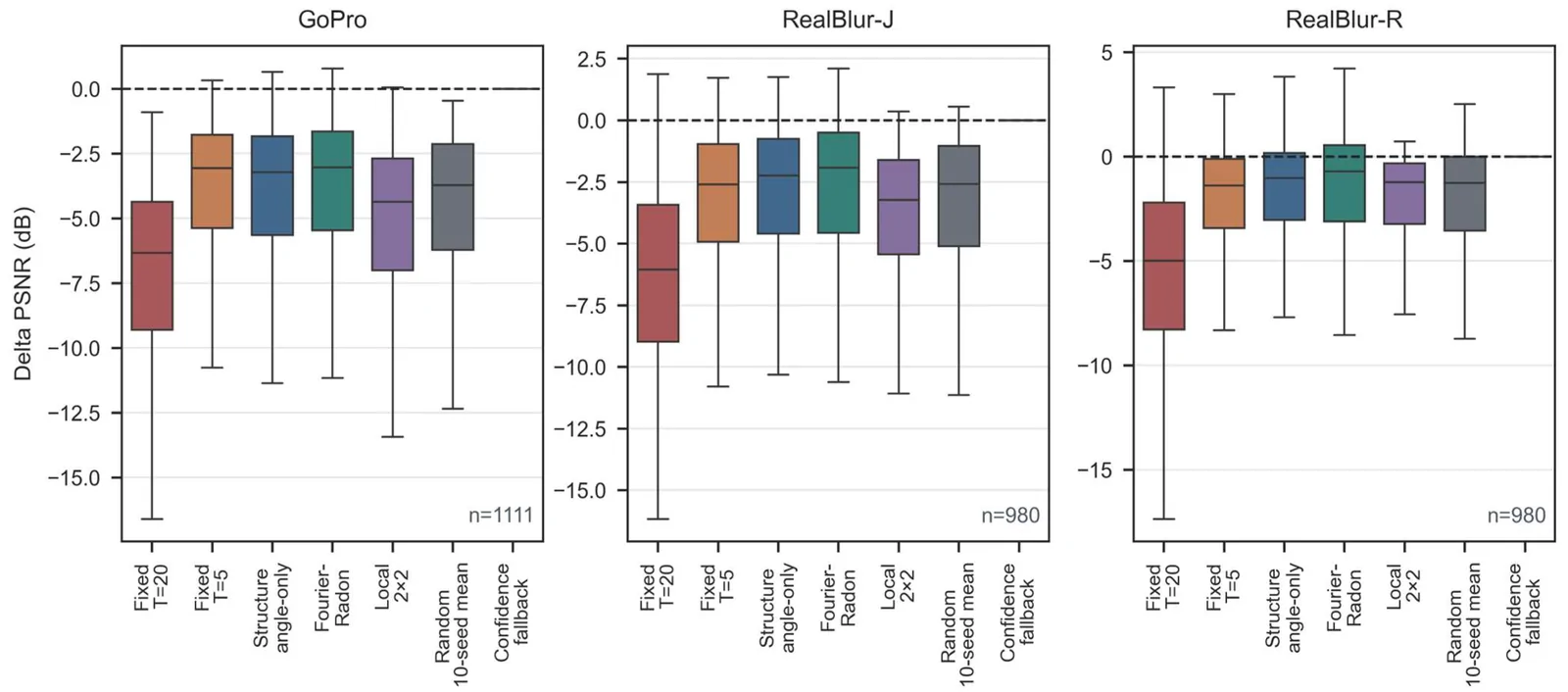
Per-image ΔPSNR distributions for fixed T = 20, fixed T = 5, structure-tensor, Fourier–Radon, joint adaptive, local, random-angle, and confidence-fallback branches under the grayscale 512 × 512 protocol. Boxes indicate the median and interquartile range; whiskers and individual density show distribution spread within GoPro, RealBlur-J, and RealBlur-R.

Across 3071 image pairs, all evaluated RL branches retained negative mean PSNR changes. Reducing fixed RL from = 20 to *T* = 5 reduced the loss. Fourier–Radon also produced a smaller mean loss than the angle-only structure tensor, but neither method produced a positive dataset mean. Table 4 summarizes the all-pair results, Figure 2 shows the per-image spread, and Supplementary Material S1 reports the complete distributions and continuity branches.

### 4.2. Multi-Seed Direction-Source Comparison

**Figure 3:**
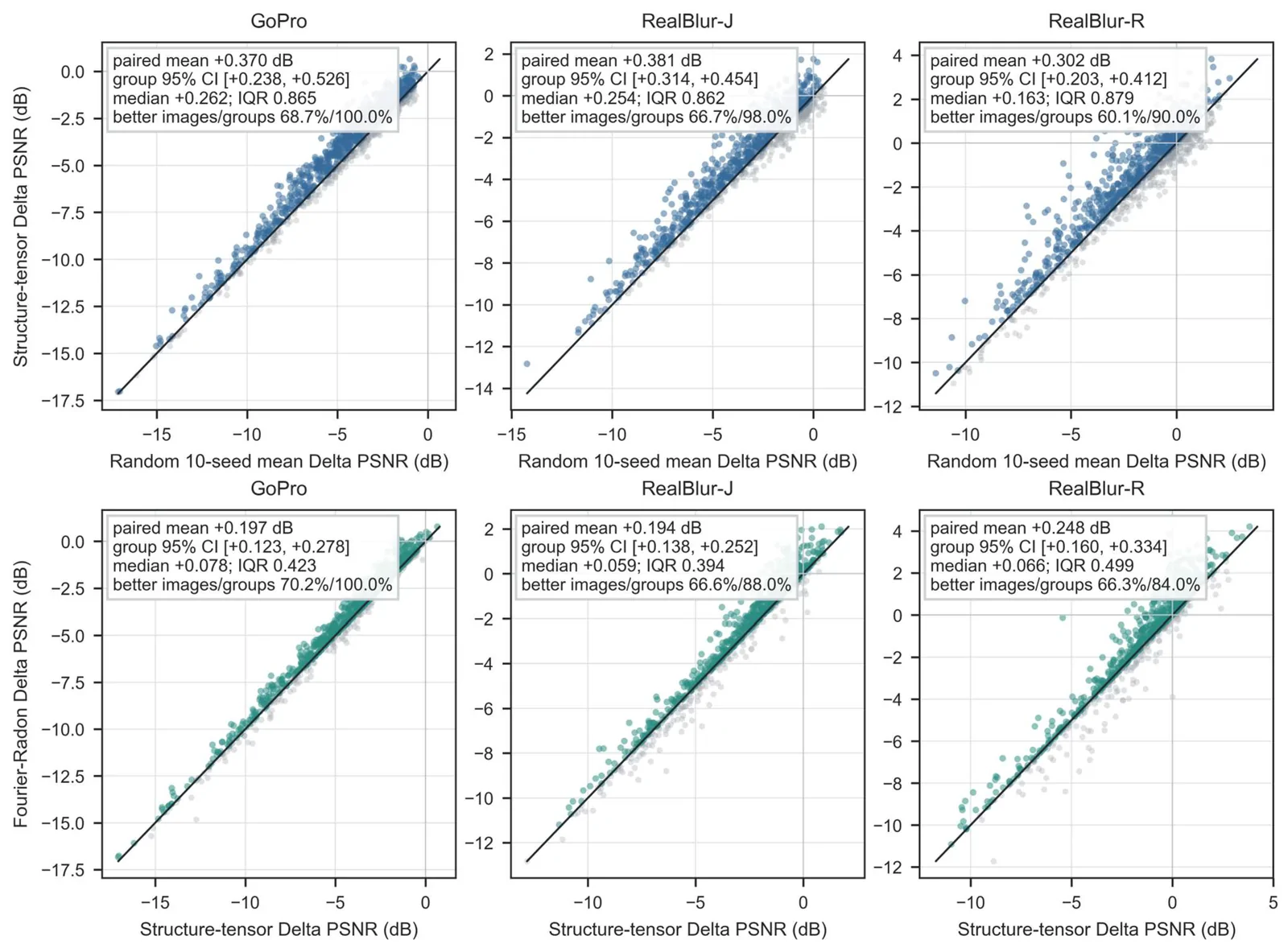
Direct paired direction-source comparisons in GoPro, RealBlur-J, and RealBlur-R. The identity diagonal denotes equal per-image ΔPSNR. Random values are the mean over 10 deterministic seeds. Reported 95% intervals resample GoPro sequences or RealBlur scenes.

Within the 3071-pair restoration tier, the structure-tensor PSNR dataset mean exceeded 10/10 random-seed dataset means in each dataset. Direct per-image paired differences remained positive after sequence/scene cluster bootstrap: +0.370 dB for GoPro, +0.381 dB for RealBlur-J, and +0.302 dB for RealBlur-R. Fourier–Radon also exceeded the structure tensor by +0.197, +0.194, and +0.248 dB. Multi-seed LPIPS supported the same relative direction-source ordering, but none of these relative advantages made restoration beneficial relative to Blur Input. Table 5 reports the direct contrasts, and Figure 3 shows the corresponding paired comparisons.

Across the 11 GoPro sequences, both primary restoration contrasts were positive in 11/11 groups. Equal-sequence mean effects were +0.352 and +0.207 dB, and exact two-sided sign-flip *p*-values were 0.000977 for both contrasts. Leave-one-sequence-out sequence means ranged from +0.297 to +0.382 dB and from +0.186 to +0.225 dB. These checks use 11 inferential units and do not convert the design into a large-cluster study.

On the GoPro dataset, the joint oracle yielded a ΔPSNR of +0.350 dB [−0.149, +0.767], whereas the angle-only oracle gave −1.036 dB [−2.312, +0.057], a difference of +1.386 dB [+0.682, +2.228]. On RealBlur-J, the corresponding values were +0.410 dB [+0.165, +0.634], −0.777 dB [−1.251, −0.351], and +1.187 dB [+0.895, +1.505]; on RealBlur-R, they were +0.616 dB [+0.384, +0.836], −0.259 dB [−0.728, +0.163], and +0.875 dB [+0.591, +1.203]. Each oracle selected the best among 900 candidate kernels per image, so these values represent selection-optimistic upper bounds. These results indicate that joint angle-and-length selection can recover cases missed by the L = 13 angle-only oracle, but they do not provide a usable estimator, nor do they separate spatially varying blur from ground-truth-assisted model selection.

### 4.3. Confidence Discrimination

**Figure 4:**
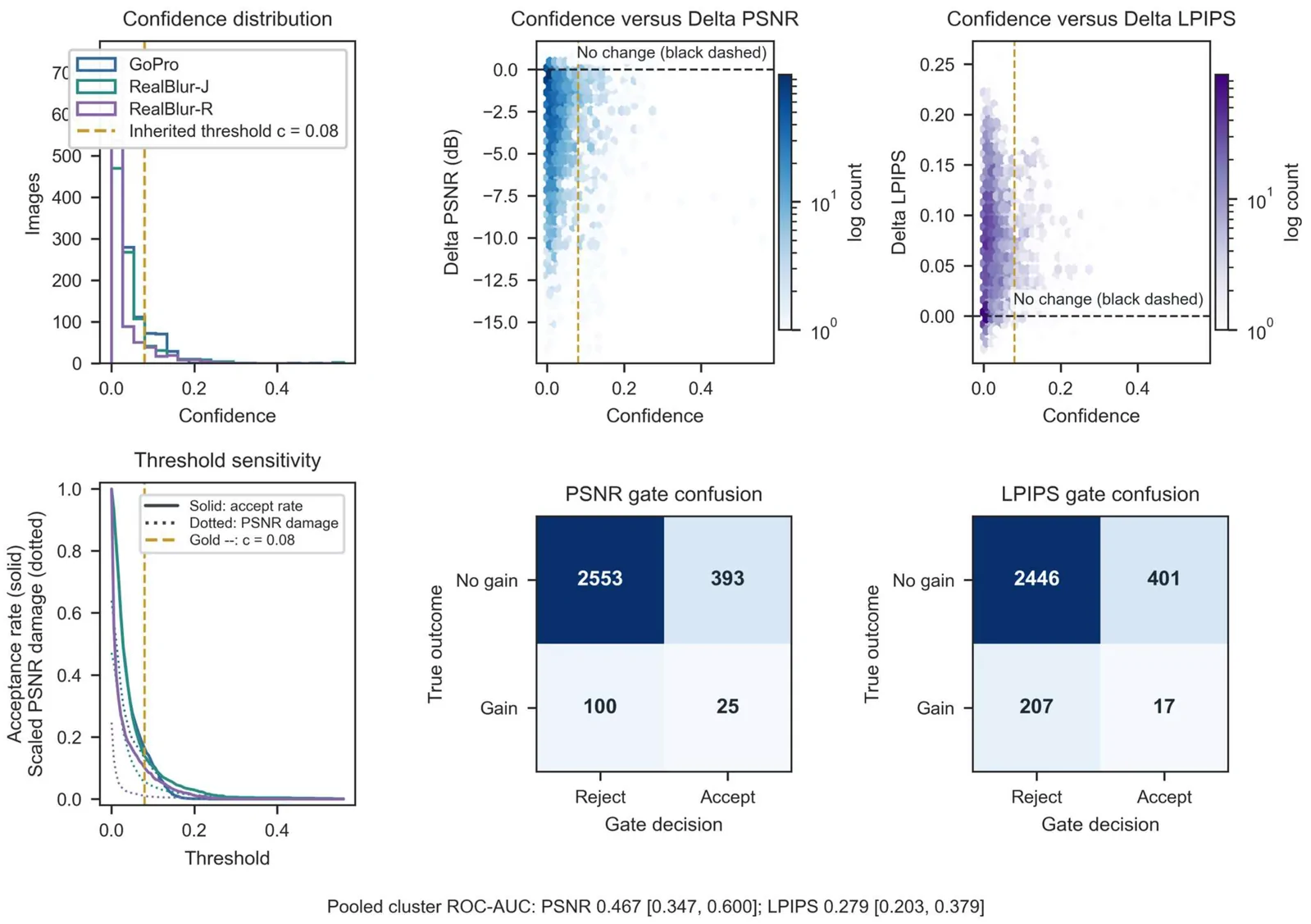
Confidence diagnostics for the ungated joint angle–length branch. Blue, teal, and purple denote GoPro, RealBlur-J, and RealBlur-R, respectively. The vertical line marks the inherited diagnostic operating point *c* = 0.08; gain labels indicate ΔPSNR > 0 or ΔLPIPS < 0 relative to Blur Input. In the threshold-sensitivity panel, solid curves show the acceptance rate and dotted curves show scaled PSNR damage (−mean ΔPSNR/8, clipped to [0, 1]). Gold dashed vertical lines mark the inherited diagnostic threshold *c* = 0.08; black dashed horizontal lines mark zero change relative to Blur Input. The footer reports pooled cluster-bootstrap intervals only. Dataset-specific AUCs are descriptive, and GoPro PSNR contains one positive image; therefore, macro AUC is not presented as headline evidence. The operating point was neither optimized nor independently validated.

In the 3071-pair tier, confidence discrimination was strongly dataset-dependent. Dataset-specific PSNR ROC-AUC values were 0.698, 0.758, and 0.612 for GoPro, RealBlur-J, and RealBlur-R, whereas pooled PSNR ROC-AUC was 0.467 [0.347, 0.600]. Pooled LPIPS ROC-AUC was 0.279 [0.203, 0.379], indicating inverse ranking under the intended interpretation. Positive counts, prevalence, average precision, and valid/excluded bootstrap replicates are reported in Supplementary Material S1; Figure 4 retains the score and threshold plots.

### 4.4. Full-Size and Boundary Sensitivity

**Figure 5:**
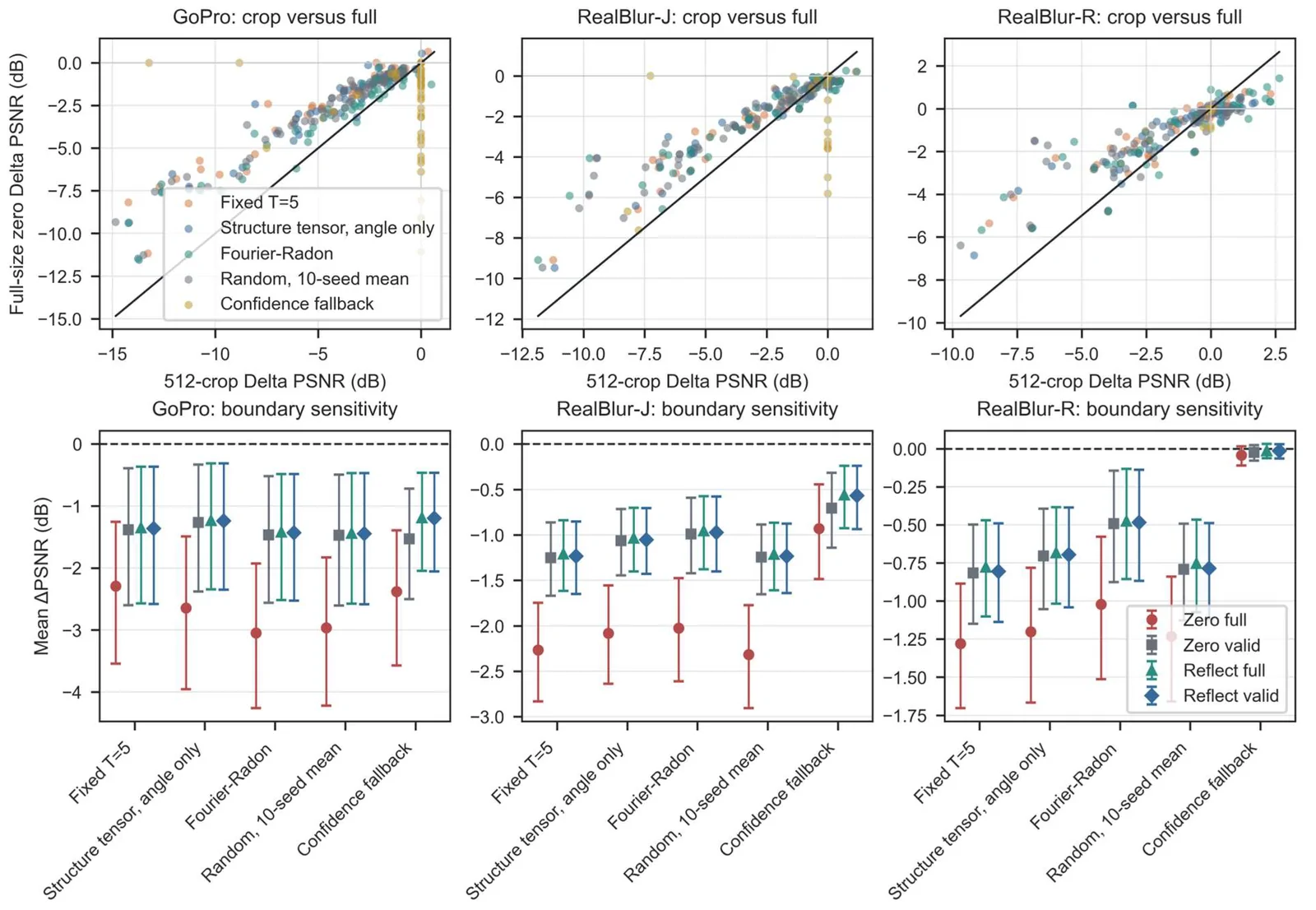
Crop, full-size, and boundary sensitivity for the previously fixed 155-image subset. The diagonal denotes equality between 512-crop and full-size ΔPSNR. Error bars are 95% sequence- or scene-cluster bootstrap intervals. Zero, reflection, full-image, and valid-interior conditions use the same images and method definitions.

On the previously fixed 155-image sensitivity subset, zero padding increased the full-image restoration loss and the border RMSE. Reflection padding and valid-interior scoring reduced the edge penalty, but all ungated direction branches retained negative mean changes relative to blurred input. The direct structure-minus-random advantage remained positive for GoPro and RealBlur-J under zero/reflection full and valid scoring, whereas RealBlur-R intervals crossed zero. Figure 5 shows the comparison and Supplementary Material S1 reports all boundary estimates.

The fixed subset covered every available sequence and scene group. Table 6 compares its composition and Blur Input distributions with the full restoration tier and reports baseline feature statistics. Standardized differences in PSNR and SSIM were small (absolute values ≤ 0.129); however, this descriptive agreement does not suffice to establish independent representativeness or adequate confirmatory power.

### 4.5. Kernel-Length Sensitivity

On the same 155-image subset, reducing the tested kernel size lowered the mean PSNR loss across the fixed, structure-tensor, and Fourier–Radon directions (Table 7). At *L* = 5 the output was closest to the Blur Input, yet most dataset–direction confidence intervals remained below zero; the GoPro Fourier–Radon intervals for *L* = 5 and *L* = 9 crossed zero without exhibiting a positive mean effect. These results establish sensitivity to the tested kernel extent, rather than an independent or dominant causal role of length mismatch.

Complete Wiener balance, boundary, and full-size RGB sensitivity records are retained in Supplementary Material S1. They constrain scope and alternative explanations but are not treated as primary evidence for feature-level reliability.

### 4.6. Qualitative Failure Examples

**Figure 6:**
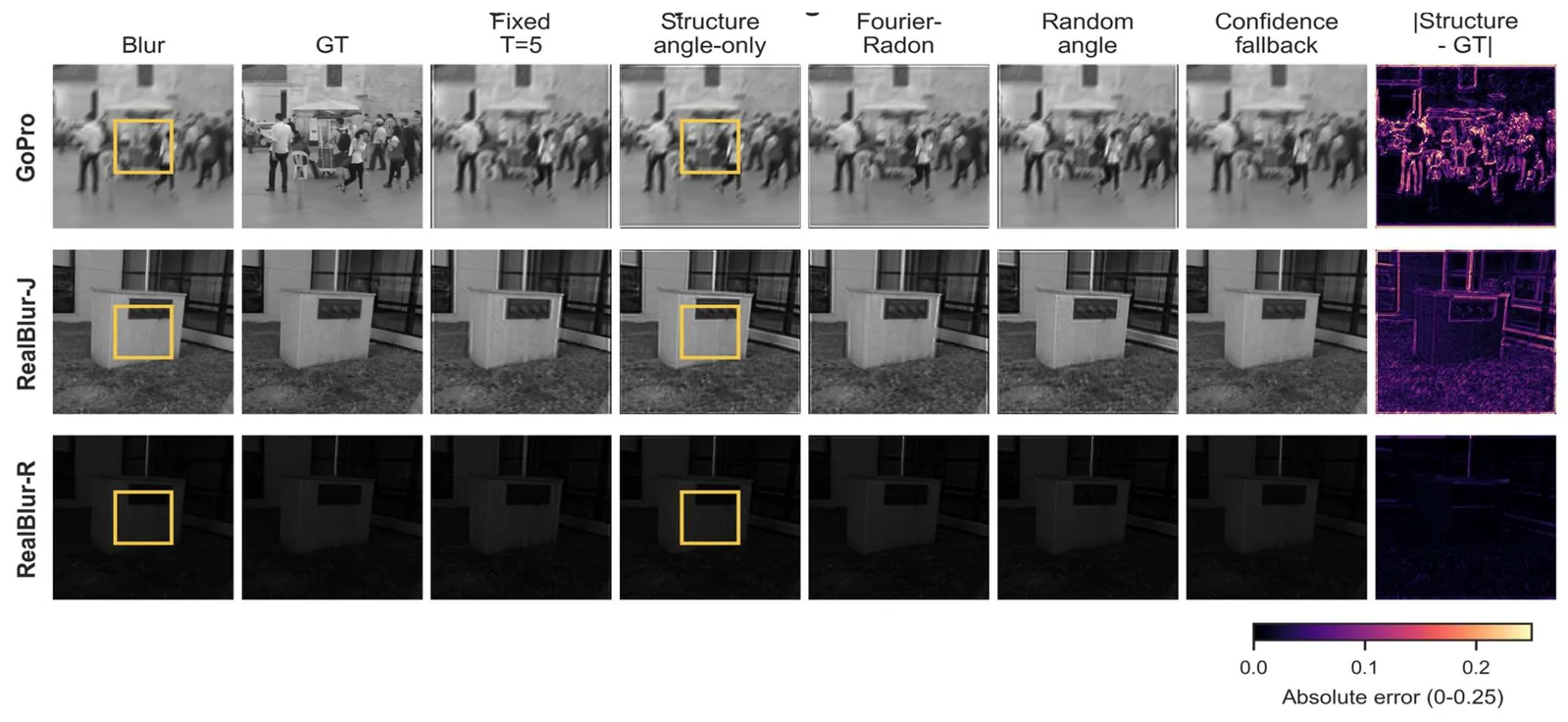
Representative full-image failures for GoPro, RealBlur-J, and RealBlur-R under the grayscale evaluation protocol.

Figure 6 presents original-intensity full-image failure examples for the three public datasets, with the central region of interest (ROI) marked on Blur and structure-tensor panels. Larger original-intensity ROIs are moved to Supplementary Material S1 to avoid an unreadable six-row composite.

Columns show Blur Input, ground truth, fixed T = 5, structure-tensor angle-only, Fourier–Radon, one deterministic random-angle realization, confidence fallback, and the structure-tensor absolute-error map. Yellow boxes mark the ROI shown in Supplementary Material S1. Original intensity is retained; no display normalization contributes to metrics. Error maps share the 0–0.25 color scale. Image samples were adapted from the GoPro dataset (Nah et al. [12], CC BY 4.0) and the RealBlur dataset (Rim et al. [13], CC BY 4.0). Cropping, restoration outputs, error maps, and panel assembly were produced by the authors.

### 4.7. Random-Direction, Failure-State, and Feature-Protocol Sensitivity

**Figure 7:**
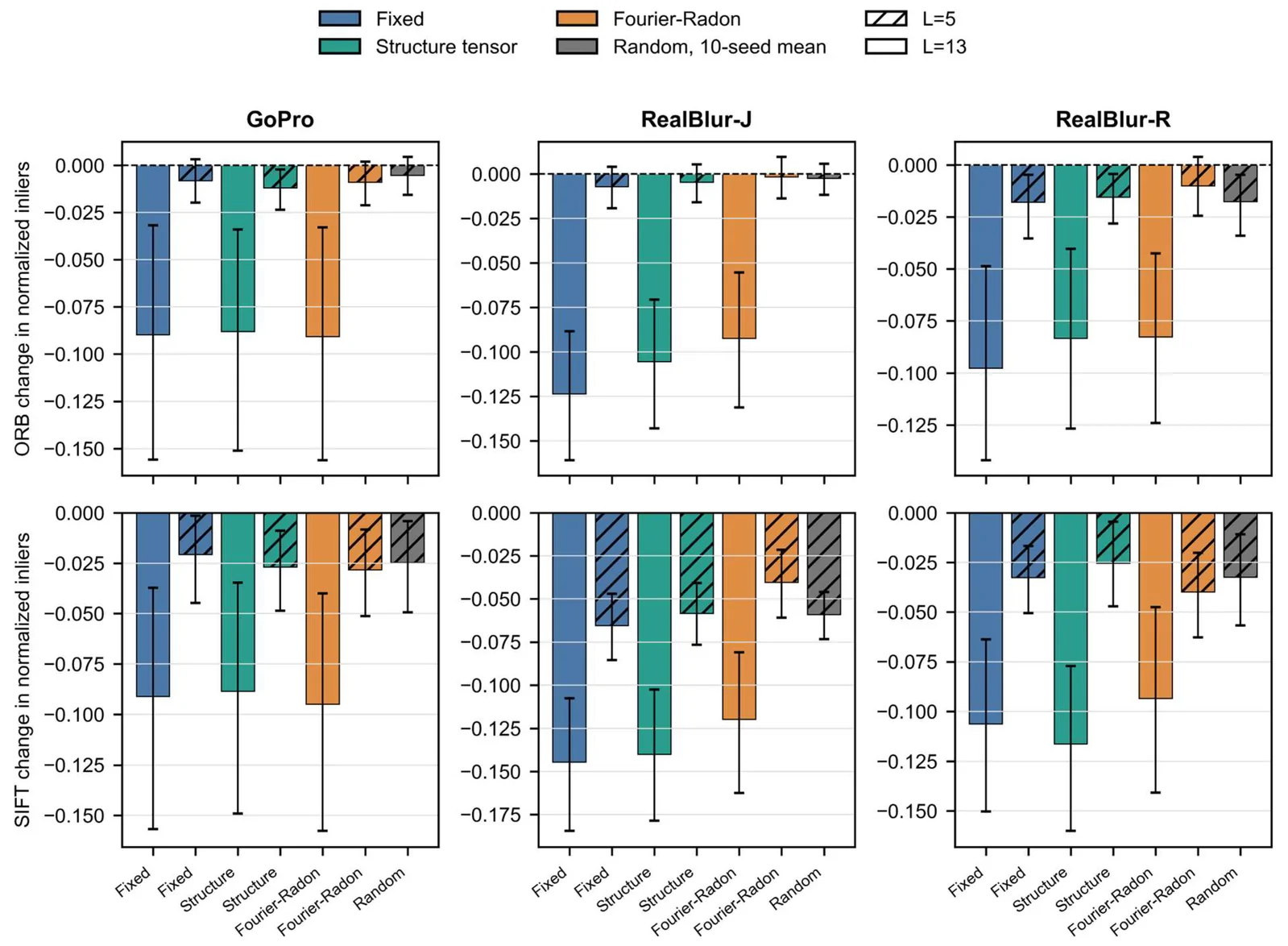
Kernel-length, direction-source, and detector sensitivity on the previously fixed 155-image subset. Blue, green, orange, and gray denote fixed, structure-tensor, Fourier–Radon, and 10-seed random directions, respectively; solid bars denote L = 13 and hatched bars denote L = 5. Error bars are sequence- or scene-cluster 95% CIs. Random seeds are averaged within image before inference.

Within the exploratory 155-image feature tier, the *L* = 5 random-angle extension altered the interpretation of the direction source (Table 8). The 10-seed random mean produced ORB changes of −0.005, −0.002, and −0.018 in GoPro, RealBlur-J, and RealBlur-R, respectively; the first two intervals crossed zero, whereas the RealBlur-R interval remained negative. The SIFT random-mean changes were negative in all datasets. Direct paired contrasts did not show a consistent advantage for any estimated direction. Fourier–Radon exceeded the random mean only for RealBlur-J SIFT (+0.019 [0.006, 0.033]), while the structure tensor was lower than the random mean for GoPro ORB (−0.007 [−0.012, −0.002]). The remaining homography contrasts all contained zero. Figure 7 summarizes the corresponding kernel-length, direction-source, and detector contrasts. The revision-defined 12-comparison family used group-level sign-flip tests and BH-FDR adjustment; no contrast remained significant at q < 0.05, with the smallest adjusted values being q = 0.109 for RealBlur-J SIFT Fourier–Radon and q = 0.199 for GoPro ORB structure tensor. Effect sizes and cluster-level confidence intervals therefore remain the primary evidence, and the broader parameter grid is descriptive.

Normalized inliers were interpreted together with the raw components (Table 9). The Blur Input mean normalized inlier values were 0.255, 0.281, and 0.285 for GoPro, RealBlur-J, and RealBlur-R, respectively. For the evaluated L = 5 ORB-homography branches, changes relative to these baselines ranged from −0.6% to −6.3%. In RealBlur-R, raw inlier gains of 33.2 to 36.6 had cluster confidence intervals above zero, while candidate keypoint counts increased by 107.7 to 128.9; by contrast, most GoPro and RealBlur-J raw inlier intervals included zero. A lower normalized value therefore does not equate to fewer raw geometrically consistent correspondences. Complete good-match and inlier-ratio intervals are provided in Supplementary Material S1.

The ORB cap audit exposed strong dataset dependence. In GoPro, 100% of reference and candidate images reached 2000 keypoints for Blur Input, every deterministic L = 5 branch, and every random seed; $q_{norm}$ was therefore raw inliers/2000 throughout this dataset. In RealBlur-J, 90% of references reached the cap, candidate saturation ranged from 78% to 92%, and denominator saturation ranged from 76% to 88% across Blur Input and deterministic L = 5 branches; 90.2% of random seed–image pairs were capped. In RealBlur-R, only 14% of references and 10–16% of deterministic candidates were capped, with denominator saturation of 10–14%; random seed–image saturation was 15.8%. Consequently, normalized inlier changes must be interpreted as endpoint-dependent measurements whose denominator behavior differs across datasets, not as a detector-independent measure of geometric evidence.

The cap sequence continued through nfeatures = 24,000. At the stopping point, the maximum reference and candidate cap proportions were 23.6% and 12.7%; the frozen rule stopped because the effects of consecutive limits satisfied the 0.005 stability criterion without sign reversal. The final mean Δq values for structure tensor/Fourier–Radon were −0.015/−0.016 on GoPro, −0.025/−0.023 on RealBlur-J, and −0.021/−0.014 on RealBlur-R. These changing magnitudes confirm that $q_{norm}$ depends on the ORB feature budget; raw inliers, good matches, inlier ratio, and cap proportions remain necessary for interpretation.

RealBlur-R failure-state sensitivity is summarized in Table 10. ORB baseline success was 46/50, and no deterministic or random L = 5 output changed geometry state. SIFT baseline success was 35/50. Across 500 descriptive seed–image evaluations, random L = 5 produced six success-to-failure and 18 failure-to-success events; these are not 500 independent samples. After collapsing seeds, two baseline-success images failed under at least one seed and four baseline-failure images were rescued under at least one seed. Inferential effects were averaged across the 10 seeds within each image before cluster resampling. The baseline-success-only sensitivity retained negative SIFT intervals and negative ORB intervals for fixed, structure, and random, while the Fourier–Radon ORB interval crossed zero.

Parameter sensitivity was summarized by effect ranges rather than votes across configurations. The 18 conditions per detector share the same images and overlapping ratio, RANSAC, and geometry settings; they are correlated sensitivity conditions, not independent replications. For example, RealBlur-J ORB Fourier–Radon L = 5 ranged from −0.002 to +0.007 with a median of +0.003, while the random mean ranged from −0.002 to +0.004 with a median near zero. The complete heatmap has been moved to Supplementary Figure S3 so that the main text remains focused on direction, kernel length, raw/normalized evidence, confidence, and failure states.

Confidence ranking remained uncertain on the feature subset (Table 11). Cluster-bootstrap ROC-AUC intervals were [0.500, 0.908], [0.545, 0.914], and [0.375, 0.936]; AP intervals were also broad. At the historical *c* = 0.08 operating point, 30/39, 11/16, and 6/8 accepted outputs had negative primary-endpoint changes, while 2, 4, and 4 positive outputs were rejected. The operating point was neither optimized nor independently validated. Supplementary threshold curves are descriptive diagnostics and do not define a selector.

### 4.8. Modern Learned-Restoration Controls

The two official learned-model controls successfully separated the RL-specific negative result from general restoration preprocessing. On the GoPro dataset, NAFNet yielded a ΔPSNR-Y of +7.758 dB [CI: +6.476, +9.026] and an ORB normalized inlier change Δq of +0.457 [CI: +0.353, +0.553]; Restormer yielded a ΔPSNR-Y of +7.581 dB [CI: +6.389, +8.861] and an ORB Δq of +0.450 [CI: +0.351, +0.546]. Table 12 reports the pixel-level, raw-feature, normalized-feature, and geometry-state changes across all three datasets. The RealBlur values are cross-dataset sensitivity results because neither checkpoint was tuned on those data.

### 4.9. Locked Regularization and Stopping Controls

Calibration selected τ = 4 for unregularized RL and λ = 0.01, τ = 4 for RL-TV. The selected λ was the upper endpoint of the frozen grid and was not expanded after inspection. On the 120-image group-disjoint validation partition, all six dataset-branch mean PSNR changes were negative, with cluster confidence intervals entirely below zero. ORB Δq intervals included zero in every cell, whereas RealBlur-R raw-inlier changes were positive for both branches. Table 13 summarizes the locked validation results.

### 4.10. Ground-Truth Task-Level RGB-D Odometry

**Figure 8:**
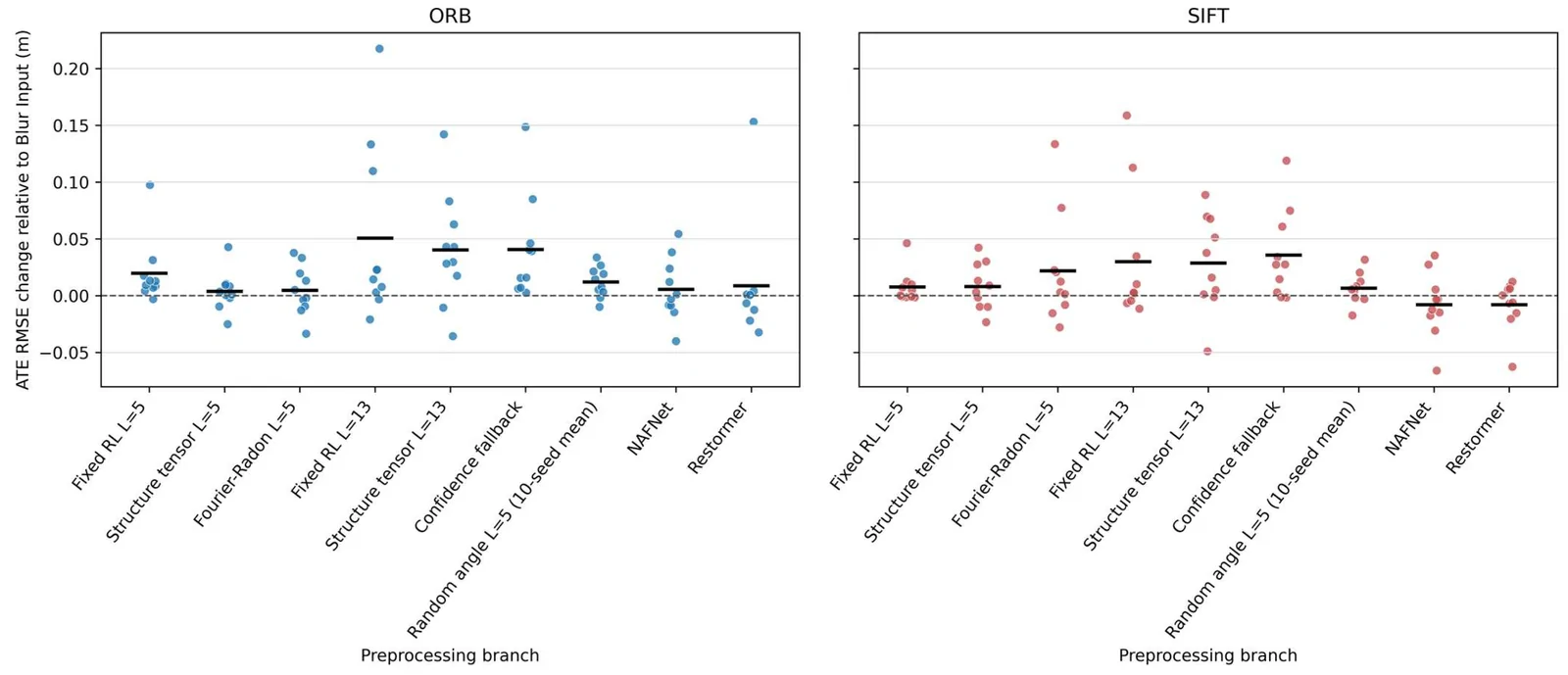
Sequence-level RGB-D odometry effects on the ten frozen TUM RGB-D sequences. Each point represents the processed-minus-Blur Input ATE RMSE for a single sequence; black horizontal bars are sequence means, and the dashed line indicates no change. Random-angle values were averaged over ten seeds within each sequence before plotting. Negative values favor preprocessing. ORB was the primary detector, and SIFT was secondary. Synthetic exposures were used only for this task-level sensitivity analysis.

For ORB, the mean ATE change ranged from +0.004 m (structure-tensor RL, *L* = 5) to +0.051 m (fixed RL, *L* = 13). Bootstrap intervals were entirely below zero for 0/9 branches, entirely above zero for 5/9 branches, and crossed zero for the remaining branches. For SIFT, the mean ATE change ranged from −0.008 m (NAFNet) to +0.036 m (confidence fallback). Bootstrap intervals were entirely below zero for 0/9 branches, entirely above zero for 4/9 branches, and crossed zero for the rest. Table 14 reports all branches against the same sequence-level Blur Input baseline. Figure 8 visualizes the sequence-level ATE differences for each detector–branch combination.

All sequence effects in Table 14 use ten independent sequence units rather than frames. PnP failures are retained in the trajectory through identity updates, so the changes reflect both correspondence quality and transitions between successful and failed motion estimates. Complete raw sequence metrics, RPE, exact tests, wild-cluster intervals, leave-one-sequence-out ranges, failure counts, coverage, and runtime diagnostics are provided in Supplementary Table S35.

### 4.11. Quantitative Distortion and Precision

Table 15 presents three distortion changes in the edge and gradient domains for each *L* = 5 branch. Relative to Blur Input, all nine dataset–method cells showed positive mean changes in edge-envelope violation and Laplacian residual energy; mean gradient distortion also increased in every cell, but the group-level 95% confidence intervals for the three GoPro branches and two RealBlur-J estimated branches included zero. The association between distortion and the normalized inlier endpoint was most pronounced in GoPro and RealBlur-J: the Spearman correlation between gradient distortion and Δ$q_{norm}$ was −0.565 [−0.753, −0.274] for the GoPro Fourier–Radon branch and −0.670 [−0.829, −0.436] for the RealBlur-J Fourier–Radon branch. In RealBlur-R, the associations were weaker and less precise. These results indicate that gradient redistribution is associated with feature-endpoint decline in some datasets, but they do not establish causation, nor do they fully account for the divergence between raw and normalized inliers observed in RealBlur-R.

Table 16 reports the estimation precision for the primary endpoint—the change in normalized ORB-homography inliers—on the fixed subset. Across the nine *L* = 5 dataset–method cells, the 95% confidence interval half-widths ranged from 0.0109 to 0.0162, and the 80% minimum detectable effect (MDE) ranged from 0.0156 to 0.0230 normalized inlier units, corresponding to 5.5–8.1% of each dataset’s Blur Input mean. Effects smaller than this range therefore cannot be precisely estimated in this subset. When a confidence interval crosses zero, this result cautions against directly concluding equivalence; at the same time, it neither negates the observed direction of change nor implies that adequate confirmatory power has been established.

## 5. Discussion

### 5.1. Direction Information Is Present but Insufficient

Across all three datasets, the estimated blur directions retained relative PSNR information. Exact randomization tests across the 11 sequences supported both GoPro restoration contrasts. Table 5 summarizes the direct contrasts between direction sources. The angle-only oracle (*L* = 13) remained negative, whereas the ground-truth-assisted joint angle–length oracle was substantially higher. This result indicates that joint angle–length selection is important within the tested PSF family. However, the reference-based selection among 900 candidates prevents a directly deployable or causal interpretation. The globally estimated branches still remained below Blur Input.

The Fourier–Radon direction exceeded the structure tensor by 0.194–0.248 dB, with direct group confidence intervals entirely above zero. In synthetic calibration, the direct mapping yielded a mean axial error of 11.97°, whereas the 90-flipped mapping produced an error of 78.03°; this result excludes an obvious coordinate-convention reversal. It does not, however, establish accuracy on real, spatially varying motion blur—such images lack directional ground truth and differ in PSF, noise, texture, and boundary conditions.

### 5.2. Kernel Length Changes the Feature-Level Failure Boundary

The restoration experiments showed that *L* = 5 caused less damage than *L* = 13. The ORB feature budget had to reach 24,000 before triggering the frozen stopping rule, and $q_{norm}$ changed substantially across different feature limits. Raw inliers could increase while $q_{norm}$ decreased, because the normalization denominator (the smaller of the candidate and reference keypoint counts) changed. Shorter kernels reduced the perturbation, but given the influence of detector caps and denominator behavior, a detector-independent claim of “feature harm” cannot be made.

### 5.3. Plausible Path from PSF Mismatch to Endpoint Divergence

The impact of a global point-spread function (PSF) mismatch extends beyond PSNR. Measurements showed that all *L* = 5 branches increased mean edge-envelope violation and edge-band Laplacian residual energy relative to the Blur Input. In GoPro and most RealBlur-J branches, gradient distortion was negatively associated with normalized ORB-homography inliers, whereas the confidence intervals in RealBlur-R were broad and mostly included zero. This pattern is consistent with RL redistributing high-frequency gradients and inducing edge ringing—ringing alters detector responses without necessarily increasing geometrically consistent matching opportunities. However, this mechanism does not fully explain the observation in RealBlur-R where raw inliers rose while $q_{\mathrm{norm}}$ fell, because denominator expansion and geometry-state changes also co-occurred. These distortion metrics were selected post hoc, and ringing amplitude was not isolated experimentally; therefore, the results reveal a quantitative association rather than a causal mechanism.

### 5.4. Confidence Has Limited Ranking Information but Poor Threshold Utility

Feature ROC-AUC point estimates exceeded 0.69, but group-bootstrap confidence intervals were broad and positive outcomes were uncommon. At *c* = 0.08, false accepts dominated in all three datasets. The confidence branch demonstrates the failure of the tested score to support reliable acceptance decisions at the inherited operating point. It should not be interpreted as a calibrated selector.

### 5.5. Boundary Effects Are Real but Not Sufficient

Zero padding increased border error relative to reflection padding, confirming an edge contribution. Reflection padding and valid-interior scoring reduced the penalty. However, ungated branches remained below Blur Input. Under both full and valid-interior scoring, the advantage of the structure tensor over random directions persisted in GoPro and RealBlur-J, whereas the direct contrast intervals for RealBlur-R crossed zero. Boundary handling affects the effect size but does not reverse the main restoration result.

### 5.6. Implications for Paired Camera-Image Feature Evaluation

RealBlur-R exhibited the clearest endpoint disagreement: several RL branches increased raw ORB inliers while their normalized inlier count $q_{\mathrm{norm}}$ decreased. The locked discrepancy control reproduced this split—raw inlier changes were positive, whereas the $q_{\mathrm{norm}}$ confidence intervals crossed zero. Under the same evaluation pipeline, NAFNet and Restormer improved multiple pixel and feature endpoints. Restoration effects should therefore be reported per endpoint rather than collapsed into a single “feature harm” label.

### 5.7. What the Controls Clarify

The control experiments rule out several simple explanations. Reflection padding and valid-interior scoring exclude zero padding as the sole cause. Exact sequence randomization separates frame count from group consistency. The expanded ORB feature cap reveals the dependence on normalization. The joint oracle demonstrates the potential of reference-assisted angle–length selection. Locked discrepancy stopping and RL-TV reduce some losses but do not yield validation-set PSNR gains. The two learned controls confirm that this negative pattern is specific to the tested estimated global RL pipeline.

### 5.8. Translation to a Ground-Truth Motion Endpoint

The RGB-D experiment introduces a ground-truth motion endpoint, but still-image metrics are not universal task proxies. Rankings differed between ORB and SIFT and between ATE and PnP success. Gains in PSNR, raw inliers, or normalized inliers alone did not predict trajectory behavior. This sequence-level result applies only to the declared synthetic exposure and RGB-D PnP chain.

This task tier separates two previously conflated claims. It tests whether selected front ends alter ground-truth-referenced camera-motion estimation, whereas the 155-image analysis explains changes in correspondences and geometry states. Agreement across tiers strengthens a branch-specific interpretation; disagreement shows that the choice of endpoint matters.

## 6. Limitations

The restoration analysis used 3071 grayscale 512 × 512 center crops rather than official full-resolution RGB protocols. Feature results came from a fixed 155-image subset and post hoc analyses that were neither preregistered nor designed as a confirmatory study. Complete group coverage and similar baseline distributions support coverage, not independent representativeness. The new precision analysis indicates 80% MDEs of 5.5–8.1% of the Blur Input Δ$q_{norm}$ mean for the primary *L* = 5 contrasts. Smaller effects remain imprecisely estimated and should not be interpreted as equivalent to zero. The distortion metrics were also selected post hoc; their associations do not establish causal mediation. The ORB feature budget required extension to 24,000 and remained detector- and denominator-dependent. The synthetic direction study validated only the coordinate convention. *T* = 5, *L* = 5, *L* = 13, and *c* = 0.08 were fixed experimental controls, not independently calibrated operating parameters. Only λ and τ for the new RL-TV and discrepancy branches used group-disjoint internal calibration. The joint oracle remains selection-optimistic, and the RL-TV regularization weight reached the upper endpoint of its frozen grid.

This experiment evaluates trajectory-conditioned synthetic exposure on real RGB-D sequences with measured camera trajectories. It does not constitute validation on naturally blurred long-exposure video or a deployed SLAM system. The rendering omits rolling shutter, raw Bayer sampling, demosaicing, photon/read noise, optical modulation transfer function, and exposure control. The fixed RGB-D PnP pipeline has no relocalization stage. Only ten sequences were available, and ground truth contributed to exposure rendering. These data do not support claims about full SLAM reliability, sensor hardware, or real-motion-blur deployment.

## 7. Conclusions

Direction estimates carried restoration information across 3071 paired images, but the tested estimated global straight-line RL branches did not outperform Blur Input on average. In the exploratory 155-image tier, feature changes depended on kernel length, direction, detector budget, and endpoint definition; no member of the 12-comparison secondary family survived BH-FDR adjustment. The post hoc distortion measurements were consistent with gradient redistribution contributing to normalized inlier loss in GoPro and RealBlur-J, but they did not establish a causal mechanism. The primary feature analysis could resolve effects of roughly 5.5–8.1% of the Blur Input Δ$q_{norm}$ mean at 80% power under its observed group variance, so smaller effects remain uncertain. Raw and normalized inliers sometimes diverged, and the ten-sequence task analysis produced endpoint-dependent rankings. NAFNet and Restormer show that the negative result is not universal to restoration. The TUM findings concern trajectory-conditioned synthetic exposure and a fixed RGB-D PnP pipeline. They do not establish performance on naturally blurred long-exposure video, full SLAM, or sensor hardware.

## Figures and Tables

**Table 1:** Synthetic coordinate-convention calibration results; the 168 conditions are not independent estimator replications.

Estimator/Mapping	Mean Axial Error °	Median Error °	Within 15°	Recovery ΔPSNR
Fourier–Radon direct	11.97°	1.00°	85.1%	+1.963 dB
Fourier–Radon +90°	78.03°	89.00°	6.5%	−0.901 dB
Structure tensor	37.06°	23.73°	16.1%	+1.047 dB
True direction	0.00°	0.00°	100.0%	+3.045 dB

**Table 2:** Experimental settings and parameter provenance.

Item	Setting
Public paired images	GoPro 1111; RealBlur-J 980; RealBlur-R 980
All-pair representation	Grayscale; centered 512 × 512 crop
Direction-control PSF	21 × 21 support; *L* = 13 pixels for direction-source isolation (fixed control; not independently calibrated)
Direction-control iterations	*T* = 5 low-iteration control; *T* = 20 over-iteration stress branch (fixed controls; not optimized)
Fixed direction	0° in image coordinates
Fourier–Radon	Hann window; log power spectrum; side 256; 1-degree grid
Direction calibration	168 known-blur conditions; direct and 90-degree mappings
Random control	10 seeds: 20260701–20260710; SHA-256 image-ID mapping
Full-size subset	Pre-run fixed stratified: 55 + 50 + 50 images
Valid interior	10-pixel border excluded
Cluster bootstrap	10,000 replicates; seed 20260711; sequence/scene clusters
Confidence bootstrap	5000 replicates; seed 20260711; sequence/scene clusters
Confidence threshold	*c* = 0.08 inherited diagnostic operating point; neither optimized nor independently validated; not proposed for deployment
Wiener balance	Nominal untuned 0.015 retained for continuity; sensitivity 0.001–0.1
Camera-feature subset	Same previously fixed 155 full-size grayscale pairs; *L* = 5 lower-disturbance sensitivity control and *L* = 13 direction-source/length control
ORB/RANSAC	Primary: ORB, ratio 0.75, homography, 3 pixels
Edge/feature inference	ORB/SIFT; homography/affine; ratio 0.70/0.75/0.80; RANSAC 2/3/5 pixels; edge tolerance 1/2/3
Feature bootstrap	10,000 replicates; seed 20260714; sequence/scene clusters
Evidence hierarchy	Restoration: 3071 pairs; feature and learned controls: fixed 155-image subset
Modern control	Official GoPro-pretrained Restormer and NAFNet; full-size RGB; no retraining or dataset-specific tuning
ORB cap sensitivity	nfeatures = 1000 to 24,000; +4000 extension after 8000; frozen cap/effect-stability stopping rule
GoPro small-cluster inference	Exact $2^{11}$ Rademacher sign-flip randomization of sequence-level mean effects
Joint oracle	Fixed 155; grayscale 512 crop; reflection; *T* = 5; five lengths × 180 integer angles; GT-assisted PSNR selection
RL stopping controls	35-image calibration/120-image group-disjoint validation; *L* = 5 Fourier–Radon; only λ and τ were internally calibrated and then locked

**Table 3:** Analysis hierarchy, inferential units, and multiplicity treatment. The feature and task tiers are exploratory sensitivity analyses rather than fully powered confirmatory studies.

Tier	Data	Inferential Unit	Principal Endpoint	Uncertainty/Multiplicity
Restoration	3071 paired images	Sequence/scene	ΔPSNR and direct direction contrasts	Cluster CIs; exact GoPro checks
Coordinate calibration	168 correlated synthetic conditions	Condition (descriptive)	Axial angular error	Convention check; no replication claim
Feature sensitivity	Fixed 155-image subset	Sequence/scene	Primary $q_{norm}$; raw and state endpoints	BH-FDR for 12 fixed secondary contrasts; grids exploratory
Stopping/RL-TV	35 calibration + 120 validation images	Disjoint sequence/scene groups	Locked $q_{norm}$ and ΔPSNR	Internal calibration-validation only
Task sensitivity	10 TUM RGB-D sequences	Sequence	ATE RMSE	Bootstrap, exact sign-flip, leave-one-out
Post hoc distortion and precision	Fixed 155-image subset	Sequence/scene	GT-referenced distortion; ORB q precision	10,000 cluster bootstrap for associations; group t CI and 80% MDE

**Table 4:** All-pair restoration summary under the grayscale 512 × 512 protocol. Cells report mean ΔPSNR [sequence/scene-cluster 95% CI]. Complete medians, IQRs, improved-image/group proportions, local/adaptive results, and the nominal untuned Wiener continuity branch are provided in Supplementary Material S1.

Dataset	Fixed T = 20	Fixed T = 5	Structure	Fourier–Radon	Random mean	Confidence
GoPro	−7.277 [−8.883, −5.911]	−4.069 [−5.402, −2.999]	−4.224 [−5.559, −3.093]	−4.027 [−5.403, −2.916]	−4.595 [−5.858, −3.438]	−1.062 [−2.341, −0.279]
RealBlur-J	−6.459 [−7.096, −5.846]	−3.197 [−3.673, −2.731]	−2.915 [−3.407, −2.434]	−2.720 [−3.255, −2.211]	−3.295 [−3.804, −2.813]	−0.438 [−0.670, −0.239]
RealBlur-R	−5.518 [−6.388, −4.703]	−2.070 [−2.602, −1.597]	−1.764 [−2.296, −1.271]	−1.516 [−2.081, −0.982]	−2.066 [−2.597, −1.570]	−0.084 [−0.167, −0.028]

**Table 5:** Direct paired direction-source results under the grayscale 512 × 512 protocol. Differences are formed per image before sequence- or scene-cluster bootstrap inference. The 11 GoPro sequence means are provided in Supplementary Material S1.

Dataset	Direct Contrast	Mean Paired Difference	Cluster-Bootstrap 95% CI	Median	IQR	First Method Better Images	First Method Better Groups
GoPro	Structure tensor minus random 10-seed mean (dB)	+0.370	[+0.238, +0.526]	+0.262	0.865	68.7%	100.0%
GoPro	Fourier–Radon minus structure tensor (dB)	+0.197	[+0.123, +0.278]	+0.078	0.423	70.2%	100.0%
GoPro	Random 10-seed mean minus structure tensor LPIPS	+0.0128	[+0.0047, +0.0222]	+0.0086	0.0422	62.1%	81.8%
RealBlur-J	Structure tensor minus random 10-seed mean (dB)	+0.381	[+0.314, +0.454]	+0.254	0.862	66.7%	98.0%
RealBlur-J	Fourier–Radon minus structure tensor (dB)	+0.194	[+0.138, +0.252]	+0.059	0.394	66.6%	88.0%
RealBlur-J	Random 10-seed mean minus structure tensor LPIPS	+0.0144	[+0.0103, +0.0190]	+0.0117	0.0343	66.9%	92.0%
RealBlur-R	Structure tensor minus random 10-seed mean (dB)	+0.302	[+0.203, +0.412]	+0.163	0.879	60.1%	90.0%
RealBlur-R	Fourier–Radon minus structure tensor (dB)	+0.248	[+0.160, +0.334]	+0.066	0.499	66.3%	84.0%
RealBlur-R	Random 10-seed mean minus structure tensor LPIPS	+0.0051	[+0.0030, +0.0070]	+0.0043	0.0213	61.7%	84.0%

**Table 6:** Coverage and baseline audit for the fixed 155-image feature subset. PSNR and SSIM cells show full-set/subset means. ORB and SIFT cells show subset Blur Input mean $q_{norm}$/geometry-success rate under homography, ratio 0.75, and a 3-pixel RANSAC threshold. These checks support coverage but do not establish independent representativeness.

Dataset	Full/Subset *n*	Groups	PSNR Full/Subset	SSIM Full/Subset	ORB q/Success	SIFT q/Success
GoPro	1111/55	11/11	25.86/25.92	0.779/0.774	0.255/100%	0.216/100%
RealBlur-J	980/50	50/50	26.72/26.48	0.798/0.782	0.281/100%	0.284/100%
RealBlur-R	980/50	50/50	34.87/34.75	0.932/0.931	0.285/92%	0.237/70%

**Table 7:** Kernel-length sensitivity on the previously fixed 155-image subset under the grayscale 512 × 512, reflection-boundary, *T* = 5 protocol. Cells report mean ΔPSNR [sequence/scene-cluster 95% CI]; med denotes the image-level median.

Dataset	Direction Source	*L* = 5	*L* = 9	*L* = 13	*L* = 17	*L* = 21
GoPro	Fixed 0°	−0.366 [−0.827, −0.025]; med −0.032	−1.002 [−2.008, −0.172]; med −0.093	−1.557 [−2.904, −0.414]; med −0.332	−2.007 [−3.592, −0.673]; med −0.471	−2.369 [−4.067, −0.861]; med −0.718
GoPro	Structure tensor	−0.366 [−0.860, −0.017]; med −0.029	−0.981 [−1.985, −0.161]; med −0.100	−1.522 [−2.818, −0.423]; med −0.202	−1.963 [−3.491, −0.630]; med −0.422	−2.337 [−3.984, −0.889]; med −0.599
GoPro	Fourier–Radon	−0.251 [−0.669, +0.054]; med −0.027	−0.800 [−1.853, +0.022]; med −0.069	−1.345 [−2.746, −0.122]; med −0.200	−1.766 [−3.383, −0.379]; med −0.224	−2.106 [−3.876, −0.546]; med −0.438
RealBlur-J	Fixed 0°	−0.419 [−0.635, −0.232]; med −0.223	−1.016 [−1.403, −0.672]; med −0.591	−1.477 [−1.968, −1.015]; med −1.106	−1.816 [−2.385, −1.300]; med −1.412	−2.107 [−2.682, −1.569]; med −1.462
RealBlur-J	Structure tensor	−0.367 [−0.566, −0.209]; med −0.198	−0.737 [−1.104, −0.419]; med −0.432	−1.195 [−1.680, −0.783]; med −0.428	−1.586 [−2.121, −1.107]; med −0.940	−1.900 [−2.456, −1.380]; med −1.453
RealBlur-J	Fourier–Radon	−0.265 [−0.465, −0.104]; med −0.197	−0.589 [−0.987, −0.234]; med −0.334	−1.135 [−1.679, −0.669]; med −0.239	−1.552 [−2.197, −0.947]; med −1.044	−1.947 [−2.617, −1.308]; med −1.514
RealBlur-R	Fixed 0°	−0.243 [−0.391, −0.118]; med −0.152	−0.596 [−0.884, −0.337]; med −0.200	−1.048 [−1.476, −0.663]; med −0.399	−1.444 [−1.967, −0.983]; med −0.779	−1.674 [−2.186, −1.206]; med −0.937
RealBlur-R	Structure tensor	−0.355 [−0.516, −0.234]; med −0.231	−0.578 [−0.867, −0.324]; med −0.236	−0.899 [−1.311, −0.513]; med −0.330	−1.233 [−1.734, −0.788]; med −0.697	−1.472 [−1.976, −0.992]; med −0.928
RealBlur-R	Fourier–Radon	−0.246 [−0.393, −0.126]; med −0.172	−0.346 [−0.665, −0.063]; med −0.068	−0.651 [−1.114, −0.230]; med −0.181	−1.088 [−1.667, −0.581]; med −0.470	−1.403 [−1.982, −0.881]; med −0.788

**Table 8:** *L* = 5 random-direction and direct estimated-minus-random comparisons on the previously fixed 155-image subset. Random values are changes from Blur Input after averaging 10 seeds within each image. Direct contrasts are formed per image before sequence- or scene-cluster bootstrap. Values are mean [95% CI] at ratio 0.75, homography, and 3 pixels.

Dataset	Detector	Random Mean Change	Structure—Random	Fourier–Radon—Random
GoPro	ORB	−0.005 [−0.016, +0.004]	−0.007 [−0.012, −0.002]	−0.004 [−0.010, +0.002]
GoPro	SIFT	−0.025 [−0.049, −0.004]	−0.002 [−0.013, +0.007]	−0.003 [−0.013, +0.005]
RealBlur-J	ORB	−0.002 [−0.012, +0.006]	−0.002 [−0.010, +0.005]	+0.001 [−0.006, +0.008]
RealBlur-J	SIFT	−0.059 [−0.073, −0.046]	+0.001 [−0.010, +0.013]	+0.019 [+0.006, +0.033]
RealBlur-R	ORB	−0.018 [−0.034, −0.005]	+0.002 [−0.005, +0.009]	+0.008 [−0.002, +0.018]
RealBlur-R	SIFT	−0.033 [−0.057, −0.011]	+0.007 [−0.006, +0.021]	−0.007 [−0.032, +0.016]

**Table 9:** Compact ORB-homography mechanism decomposition at L = 5. Δq, candidate-keypoint change, and raw-inlier change are mean [sequence/scene-cluster 95% CI]. Relative change uses the Blur Input q baseline. Random results average 10 seeds within image before inference.

Dataset	Method	Blur q	Δq [95% CI]	Relative	Δ Keypoints [95% CI]	Δ Raw Inliers [95% CI]	Success
GoPro	Fixed	0.255	−0.008 [−0.020, +0.003]	−3.2%	+0.0 [+0.0, +0.0]	−16.4 [−38.9, +6.5]	100.0%
GoPro	Structure	0.255	−0.012 [−0.024, −0.002]	−4.7%	+0.0 [+0.0, +0.0]	−24.1 [−46.3, −5.0]	100.0%
GoPro	Fourier–Radon	0.255	−0.009 [−0.021, +0.002]	−3.6%	+0.0 [+0.0, +0.0]	−18.2 [−42.4, +4.2]	100.0%
GoPro	Random mean	0.255	−0.005 [−0.016, +0.004]	−2.1%	+0.0 [+0.0, +0.0]	−10.7 [−32.2, +9.1]	100.0%
RealBlur-J	Fixed	0.281	−0.007 [−0.019, +0.004]	−2.5%	+58.9 [+20.2, +105.1]	−7.7 [−31.6, +15.0]	100.0%
RealBlur-J	Structure	0.281	−0.005 [−0.016, +0.005]	−1.7%	+64.8 [+22.5, +115.8]	−2.7 [−25.4, +17.7]	100.0%
RealBlur-J	Fourier–Radon	0.281	−0.002 [−0.014, +0.010]	−0.6%	+56.4 [+18.9, +101.2]	+3.4 [−21.0, +26.2]	100.0%
RealBlur-J	Random mean	0.281	−0.002 [−0.012, +0.006]	−0.9%	+62.6 [+20.8, +114.2]	+1.7 [−17.2, +18.4]	100.0%
RealBlur-R	Fixed	0.285	−0.018 [−0.035, −0.005]	−6.3%	+107.7 [+73.3, +148.1]	+33.2 [+14.3, +55.2]	92.0%
RealBlur-R	Structure	0.285	−0.016 [−0.028, −0.004]	−5.5%	+128.9 [+89.6, +175.0]	+36.6 [+17.4, +57.2]	92.0%
RealBlur-R	Fourier–Radon	0.285	−0.010 [−0.024, +0.004]	−3.5%	+109.5 [+72.0, +152.7]	+34.4 [+16.1, +55.5]	92.0%
RealBlur-R	Random mean	0.285	−0.018 [−0.034, −0.005]	−6.2%	+124.9 [+86.4, +169.5]	+36.1 [+16.1, +57.6]	92.0%

**Table 10:** RealBlur-R homography-state transitions and Blur Input-success-only sensitivity. S-S, S-F, F-S, and F-F denote baseline-to-processed states. Deterministic counts use images. Random counts use seed–image pairs descriptively only; S-F/F-S images report unique images with at least one event across 10 seeds. Inferential comparisons average seeds within image before scene-cluster resampling.

Method	Detector	Unit	S-S	S-F	F-S	F-F	S-F/F-S Images	Baseline-Success Δq [95% CI]
Fixed	ORB	image	46	0	0	4	n/a	*n* = 46; −0.020 [−0.038, −0.004]
Fixed	SIFT	image	35	0	1	14	n/a	*n* = 35; −0.049 [−0.073, −0.029]
Structure	ORB	image	46	0	0	4	n/a	*n* = 46; −0.017 [−0.031, −0.005]
Structure	SIFT	image	35	0	2	13	n/a	*n* = 35; −0.047 [−0.072, −0.025]
Fourier–Radon	ORB	image	46	0	0	4	n/a	*n* = 46; −0.011 [−0.026, +0.004]
Fourier–Radon	SIFT	image	34	1	0	15	n/a	*n* = 35; −0.057 [−0.088, −0.030]
Random	ORB	seed–image (descriptive)	460	0	0	40	0/0	*n* = 46; −0.019 [−0.038, −0.005]
Random	SIFT	seed–image (descriptive)	344	6	18	132	2/4	*n* = 35; −0.057 [−0.086, −0.032]

**Table 11:** Cluster-aware feature-confidence assessment using a fixed threshold of c=0.08. AUC and average precision were computed from the ungated joint branch. The “Valid/Excluded” column reports bootstrap replicate counts; excluded replicates lacked either outcome class. No threshold optimization was performed.

Dataset	Feature Gains	ROC-AUC [95% CI]	AP [95% CI]	Valid/Excluded	Accepted	Accepted Mean Change	False Accept	False Reject
GoPro	11/55 (20.0%)	0.692 [0.500, 0.908]	0.345 [0.129, 0.726]	9984/16	39/55	−0.149	30	2
RealBlur-J	9/50 (18.0%)	0.743 [0.545, 0.914]	0.415 [0.193, 0.772]	10,000/0	16/50	−0.204	11	4
RealBlur-R	6/50 (12.0%)	0.705 [0.375, 0.936]	0.347 [0.093, 0.760]	9988/12	8/50	−0.055	6	4

**Table 12:** Changes from Blur Input for the official GoPro-pretrained NAFNet and Restormer on the fixed 155-image subset. Values are mean [sequence/scene-cluster 95% CI] under the primary homography setting; q denotes normalized inliers. GoPro is in-domain, whereas RealBlur-J and RealBlur-R are cross-dataset sensitivity analyses.

Dataset	Model	ΔPSNR-Y	ORB Δraw	ORB Δq	SIFT Δraw	SIFT Δq	Δ Success ORB/SIFT
GoPro	NAFNet	+7.758 [+6.476, +9.026]	+913.4 [+705.8, +1106.3]	+0.457 [+0.353, +0.553]	+738.1 [+582.5, +888.0]	+0.334 [+0.258, +0.411]	+0.000 [+0.000, +0.000]/+0.000 [+0.000, +0.000]
GoPro	Restormer	+7.581 [+6.389, +8.861]	+901.0 [+702.0, +1091.7]	+0.450 [+0.351, +0.546]	+719.3 [+565.5, +869.2]	+0.325 [+0.250, +0.403]	+0.000 [+0.000, +0.000]/+0.000 [+0.000, +0.000]
RealBlur-J	NAFNet	−0.127 [−0.272, +0.045]	+120.9 [+69.8, +176.2]	+0.058 [+0.033, +0.085]	+119.5 [+88.6, +152.5]	+0.044 [+0.025, +0.064]	+0.000 [+0.000, +0.000]/+0.000 [+0.000, +0.000]
RealBlur-J	Restormer	+0.138 [−0.089, +0.389]	+234.0 [+168.1, +301.8]	+0.113 [+0.082, +0.146]	+202.9 [+154.8, +253.4]	+0.095 [+0.071, +0.119]	+0.000 [+0.000, +0.000]/+0.000 [+0.000, +0.000]
RealBlur-R	NAFNet	−0.141 [−0.377, +0.074]	+125.8 [+90.4, +163.3]	+0.081 [+0.047, +0.115]	+17.2 [+10.6, +26.7]	+0.054 [+0.019, +0.092]	+0.040 [+0.000, +0.100]/+0.140 [+0.060, +0.240]
RealBlur-R	Restormer	−0.051 [−0.267, +0.138]	+141.6 [+103.4, +181.6]	+0.111 [+0.076, +0.149]	+21.5 [+11.8, +33.8]	+0.076 [+0.043, +0.114]	+0.060 [+0.000, +0.140]/+0.100 [+0.020, +0.180]

Direct paired feature contrasts remained model- and dataset-specific. Relative to Fourier–Radon *L* = 5, NAFNet showed ORB Δq differences of +0.466 [+0.373, +0.554] on GoPro, +0.059 [+0.034, +0.086] on RealBlur-J, and +0.091 [+0.062, +0.121] on RealBlur-R. Restormer, also relative to Fourier–Radon *L* = 5, yielded differences of +0.460 [+0.368, +0.548] on GoPro, +0.115 [+0.083, +0.149] on RealBlur-J, and +0.122 [+0.087, +0.159] on RealBlur-R. These controls do not constitute a general ranking of learned methods; rather, they demonstrate that positive feature changes are achievable under the same evaluation pipeline and prevent the estimated-RL result from being generalized to all restoration front ends.

**Table 13:** Locked discrepancy-stopping and RL-TV validation results. Parameters were selected once on 35 calibration images and held fixed on 120 group-disjoint validation images. Values are mean [sequence/scene-cluster 95% CI]; T is the median stopping iteration.

Dataset	Branch	λ	τ	Median T	ΔPSNR	ORB Δq	ORB Δraw
GoPro	RL + discrepancy	0	4	2.5	−1.080 [−2.685, −0.116]	−0.021 [−0.053, +0.005]	−42.8 [−105.7, +9.1]
GoPro	RL-TV + discrepancy	0.01	4	8.5	−0.733 [−1.739, −0.081]	−0.010 [−0.032, +0.007]	−20.9 [−64.3, +14.5]
RealBlur-J	RL + discrepancy	0	4	2.0	−0.658 [−1.280, −0.187]	−0.011 [−0.034, +0.007]	−21.5 [−66.9, +14.3]
RealBlur-J	RL-TV + discrepancy	0.01	4	2.0	−0.461 [−0.912, −0.119]	−0.004 [−0.023, +0.012]	−8.6 [−45.9, +22.9]
RealBlur-R	RL + discrepancy	0	4	2.0	−0.170 [−0.308, −0.066]	−0.004 [−0.019, +0.011]	+19.8 [+3.4, +36.1]
RealBlur-R	RL-TV + discrepancy	0.01	4	2.0	−0.112 [−0.266, −0.003]	−0.003 [−0.014, +0.006]	+17.1 [+2.1, +30.4]

**Table 14:** Ground-truth-referenced RGB-D odometry after restoration preprocessing on ten frozen TUM RGB-D sequences with trajectory-conditioned synthetic exposure blur. ATE values are in meters after SE(3) alignment without scale. Differences are processed-minus-Blur Input sequence means with sequence-bootstrap 95% CIs; negative ATE and positive PnP-success differences favor preprocessing. Random-angle results are ten-seed means formed within each sequence before inference.

Detector	Branch	Blur ATE	Processed ATE	ΔATE [95% CI]	Median ΔATE	Improved Seq.	ΔPnP Success [95% CI]
ORB	Fixed RL, L = 5	0.184	0.203	+0.020 [+0.007, +0.039]	+0.011	1/10	+0.000 [+0.000, +0.001]
ORB	Structure-tensor RL, L = 5	0.184	0.188	+0.004 [−0.006, +0.015]	+0.002	3/10	+0.001 [+0.000, +0.001]
ORB	Fourier–Radon RL, L = 5	0.184	0.188	+0.005 [−0.008, +0.018]	+0.002	5/10	+0.000 [−0.000, +0.001]
ORB	Fixed RL, L = 13	0.184	0.234	+0.051 [+0.010, +0.101]	+0.019	2/10	+0.001 [+0.000, +0.001]
ORB	Structure-tensor RL, L = 13	0.184	0.224	+0.040 [+0.013, +0.071]	+0.036	2/10	+0.001 [+0.000, +0.001]
ORB	Confidence fallback	0.184	0.224	+0.041 [+0.017, +0.070]	+0.028	0/10	+0.000 [+0.000, +0.000]
ORB	Random-angle RL, L = 5 (10-seed mean)	0.184	0.196	+0.012 [+0.004, +0.020]	+0.011	2/10	+0.000 [+0.000, +0.001]
ORB	NAFNet	0.184	0.189	+0.006 [−0.010, +0.022]	−0.001	5/10	−0.001 [−0.004, +0.001]
ORB	Restormer	0.184	0.192	+0.009 [−0.013, +0.043]	+0.000	5/10	+0.001 [+0.000, +0.001]
SIFT	Fixed RL, L = 5	0.152	0.160	+0.008 [+0.001, +0.017]	+0.002	4/10	−0.000 [−0.001, +0.000]
SIFT	Structure-tensor RL, L = 5	0.152	0.160	+0.008 [−0.004, +0.020]	+0.006	4/10	−0.000 [−0.001, +0.000]
SIFT	Fourier–Radon RL, L = 5	0.152	0.174	+0.022 [−0.003, +0.053]	+0.008	3/10	−0.000 [−0.001, +0.000]
SIFT	Fixed RL, L = 13	0.152	0.182	+0.030 [+0.000, +0.068]	+0.003	3/10	+0.000 [+0.000, +0.001]
SIFT	Structure-tensor RL, L = 13	0.152	0.181	+0.029 [+0.003, +0.053]	+0.027	2/10	−0.000 [−0.001, +0.000]
SIFT	Confidence fallback	0.152	0.188	+0.036 [+0.015, +0.060]	+0.027	2/10	−0.001 [−0.002, +0.000]
SIFT	Random-angle RL, L = 5 (10-seed mean)	0.152	0.159	+0.007 [−0.001, +0.015]	+0.006	3/10	−0.000 [−0.001, +0.000]
SIFT	NAFNet	0.152	0.144	−0.008 [−0.025, +0.008]	−0.008	7/10	−0.002 [−0.004, +0.000]
SIFT	Restormer	0.152	0.145	−0.008 [−0.022, +0.003]	−0.003	5/10	+0.000 [+0.000, +0.001]

**Table 15:** Ground-truth-referenced distortion changes for the *L* = 5 branches on the fixed 155-image subset. Distortion values are group means [two-sided group-level t-based 95% CI] relative to Blur Input; positive values indicate increased distortion. The last column gives the image-level Spearman correlation between the change in gradient distortion and the change in normalized ORB-homography inliers, with a sequence/scene-cluster bootstrap 95% CI.

Dataset	Method	$\Delta_{edge\;envelope}$	$\Delta_{gradient}$	$\Delta_{Laplacian}$	ρ($\Delta_{gradient}$, $\Delta_{q_{norm}}$)
GoPro	Fixed, *L* = 5	0.0008 [0.0001, 0.0015]	0.006 [−0.018, 0.030]	0.048 [0.013, 0.082]	−0.415 [−0.627, −0.170]
GoPro	Structure tensor, *L* = 5	0.0010 [0.0001, 0.0019]	0.007 [−0.020, 0.035]	0.048 [0.017, 0.079]	−0.530 [−0.707, −0.263]
GoPro	Fourier–Radon, *L* = 5	0.0010 [0.0003, 0.0018]	0.012 [−0.015, 0.039]	0.060 [0.023, 0.098]	−0.565 [−0.753, −0.274]
RealBlur-J	Fixed, *L* = 5	0.0017 [0.0011, 0.0023]	0.017 [0.001, 0.032]	0.063 [0.039, 0.087]	−0.532 [−0.734, −0.247]
RealBlur-J	Structure tensor, *L* = 5	0.0013 [0.0009, 0.0016]	0.012 [−0.001, 0.025]	0.059 [0.040, 0.078]	−0.310 [−0.591, 0.022]
RealBlur-J	Fourier–Radon, *L* = 5	0.0013 [0.0007, 0.0019]	0.011 [−0.002, 0.025]	0.057 [0.036, 0.079]	−0.670 [−0.829, −0.436]
RealBlur-R	Fixed, *L* = 5	0.0005 [0.0003, 0.0006]	0.020 [0.009, 0.032]	0.077 [0.059, 0.095]	−0.222 [−0.507, 0.081]
RealBlur-R	Structure tensor, *L* = 5	0.0006 [0.0004, 0.0008]	0.032 [0.020, 0.045]	0.114 [0.097, 0.131]	−0.085 [−0.392, 0.232]
RealBlur-R	Fourier–Radon, *L* = 5	0.0004 [0.0003, 0.0006]	0.023 [0.011, 0.035]	0.095 [0.077, 0.114]	−0.297 [−0.552, 0.015]

**Table 16:** Group-level precision and minimum detectable effect for the primary endpoint (change in normalized ORB-homography inliers) on the fixed 155-image subset. Effects are processed minus Blur Input. The MDE is based on a two-sided test with α = 0.05, 80% power, and the observed between-sequence/scene standard deviation.

Dataset (Groups)	Method	Mean Delta $q_{norm}$	95% CI	CI Half-Width	80% MDE	MDE/Blur Mean
GoPro (*n* = 11)	Fixed, L = 5	−0.0082	[−0.0219, 0.0055]	0.0137	0.0191	7.5%
GoPro (*n* = 11)	Structure tensor, L = 5	−0.0121	[−0.0248, 0.0007]	0.0128	0.0178	7.0%
GoPro (*n* = 11)	Fourier–Radon, L = 5	−0.0091	[−0.0230, 0.0048]	0.0139	0.0194	7.6%
RealBlur-J (*n* = 50)	Fixed, L = 5	−0.0071	[−0.0190, 0.0048]	0.0119	0.0169	6.0%
RealBlur-J (*n* = 50)	Structure tensor, L = 5	−0.0047	[−0.0156, 0.0062]	0.0109	0.0156	5.5%
RealBlur-J (*n* = 50)	Fourier–Radon, L = 5	−0.0017	[−0.0138, 0.0105]	0.0122	0.0173	6.2%
RealBlur-R (*n* = 50)	Fixed, L = 5	−0.0180	[−0.0342, −0.0018]	0.0162	0.0230	8.1%
RealBlur-R (*n* = 50)	Structure tensor, L = 5	−0.0156	[−0.0278, −0.0033]	0.0123	0.0175	6.1%
RealBlur-R (*n* = 50)	Fourier–Radon, L = 5	−0.0100	[−0.0245, 0.0045]	0.0145	0.0206	7.2%

## Data Availability

GoPro, RealBlur-J, RealBlur-R, the TUM RGB-D benchmark [54], and the official Restormer and NAFNet checkpoints are available from their original public sources. Supplementary Data and Code S1 contains derived restoration, feature, and RGB-D odometry metrics; the frozen task protocol and digest; random-seed records; bootstrap, exact-test, BH-FDR, and leave-one-out outputs; figure source data; tests; and environment specifications. Third-party images, depth maps, ground-truth trajectories, restored frames, repositories, and checkpoint weights are not redistributed.

## Supplementary Materials

The following supporting information can be downloaded at: https://www.mdpi.com/article/10.3390/s26165249/s1, Supplementary Material S1 (Supplementary_Material_S1.pdf) contains Figures S1–S3, the complete supplementary-file index, and compact versions of Tables S35, S36, S37b, S38b, and S38c. Supplementary Tables S1–S38 (Supplementary_Tables_S1–S38.xlsx) contains all editable tables, lettered subtables, and source-level worksheets. Supplementary Data and Code S1 (Supplementary_Data_and_Code_S1.zip) contains the analysis code, processed CSV records, integrity tests, environment records, sanitized manifests, figure source data, task-level RGB-D records, validation scripts, and rerun instructions. Figure S1: Enlarged central regions of interest for GoPro, RealBlur-J, and RealBlur-R; Figure S2: Feature-confidence threshold diagnostics on the fixed 155-image subset; Figure S3: Correlated *L* = 5 feature-parameter sensitivity conditions across detector, geometry model, ratio-test, and RANSAC settings. Table S1: All-pair grayscale method summary; Table S2: Ten-seed random-angle summary; Table S3: Multi-seed LPIPS direct contrasts; Table S4: Confidence discrimination and bootstrap diagnostics; Table S5: Full-size boundary sensitivity; Table S6: Synthetic axial-direction calibration; Table S7: Direct direction-source contrasts; Table S8: Oracle-angle upper-bound summary; Table S9: Kernel-length sensitivity; Table S10: Wiener-balance sensitivity; Table S11: Full-size RGB sensitivity; Table S12: GoPro sequence and leave-one-sequence-out robustness; Table S13: Full ORB/SIFT baseline feature decomposition, including keypoints, good matches, raw and normalized inliers, inlier ratio, and reprojection RMSE; Table S14: Geometry success, failure counts, valid-sample counts, and failure reasons; Tables S15a and S15b: Detector, ratio-test, RANSAC-threshold, geometry-model, and edge-tolerance sensitivity; Tables S16a and S16b: Confidence acceptance/rejection decomposition and feature/PSNR ranking diagnostics; Tables S17a and S17b: GoPro sequence means, exact sign tests, and leave-one-sequence-out ranges; Table S18: Direct *L* = 5 versus *L* = 13 paired cluster comparisons; Tables S19a and S19b: *L* = 5 random-seed summaries and estimated-minus-random paired contrasts; Tables S20a and S20b: Blur Input-success-only sensitivity and four-state geometry-transition matrices; Tables S21a and S21b: Baseline, candidate, raw, normalized, relative, and success-rate feature decomposition; Tables S22a and S22b: Correlated parameter-condition effects, effect ranges, and factor-range summaries; Tables S23a and S23b: Cluster-aware feature-confidence ROC-AUC/AP intervals and complete threshold diagnostics; Tables S24a–S24j: Complete all-pair distributions, GoPro sequence-level direct contrasts, restoration-confidence diagnostics, boundary controls, Wiener sensitivity, and full-size RGB sensitivity; Table S25: Unique-image random-transition summary after collapsing the 10 seed–image evaluations within each image; Table S26: ORB $n_{features}=2000$ cap-saturation audit by dataset and *L* = 5 branch, including random seed–image sensitivity; Table S27: Fixed-subset group coverage and Blur Input distribution audit; Table S28: ORB $n_{features}=1000/2000/4000$ sensitivity for deterministic *L* = 5 branches; Table S29: Official NAFNet pixel, feature, geometry-state, and learned-versus-classical contrasts; Table S30: Exact GoPro sequence-level randomization over all $2^{11}$ assignments; Table S31: Extended ORB cap sequence through $n_{features}=$ 24,000 and the frozen stopping audit; Table S32: Official Restormer pixel, feature, geometry-state, and learned-versus-classical contrasts; Table S33: Ground-truth-assisted joint angle–length oracle contrasts and selected-parameter distributions; Table S34: RL discrepancy-stopping and RL-TV calibration/validation results; Tables S35a–S35e: Ground-truth task-level RGB-D odometry protocol, raw and seed-collapsed sequence metrics, paired effects, sequence-level inference, integrity records, and Figure 8 source data; Table S36: Fixed-subset coverage, analysis hierarchy, and Benjamini–Hochberg false-discovery-rate-adjusted secondary feature contrasts; Tables S37a–S37c: Ground-truth-referenced distortion metrics, group summaries, and feature associations; Tables S38a–S38c: Group effects and precision/minimum-detectable-effect summaries for the primary and secondary feature contrasts. Third-party public images, restored image frames, external repositories, pretrained model checkpoints, and model weights are not redistributed. Random-seed results were averaged within each image or sequence before group-level inference. The distortion analysis is post hoc and supports association rather than causal mediation, while the minimum detectable effect is reported as a precision descriptor rather than an equivalence margin.
