# Supplementary material for: Feature-Level Reliability of Directional-Kernel Richardson–Lucy Deblurring Under Kernel-Length and Direction Controls
Source: Sensors (Basel). 2026 Aug 19;26(16):5249. doi: 10.3390/s26165249 (PMC13517317; doi:10.3390/s26165249)
Supplement: Supplementary file 1 [file sensors-26-05249-s001.zip › Supplementary_Material_S1.pdf]

Supplementary Material S1

Feature-Level Reliability of Directional-Kernel Richardson–Lucy Deblurring under Kernel-Length and Direction Controls

Xiangchen Ku, Runqing Xue, and Yichen Liang

Journal: Sensors

Section: Sensing and Imaging

Affiliation: Department of Mechanical and Electronic Engineering, School of Mechatronics Engineering, Henan University of Science and Technology, Luoyang 471003, China

Corresponding author: Xiangchen Ku (kuxch@haust.edu.cn)

Supplementary Figure S1

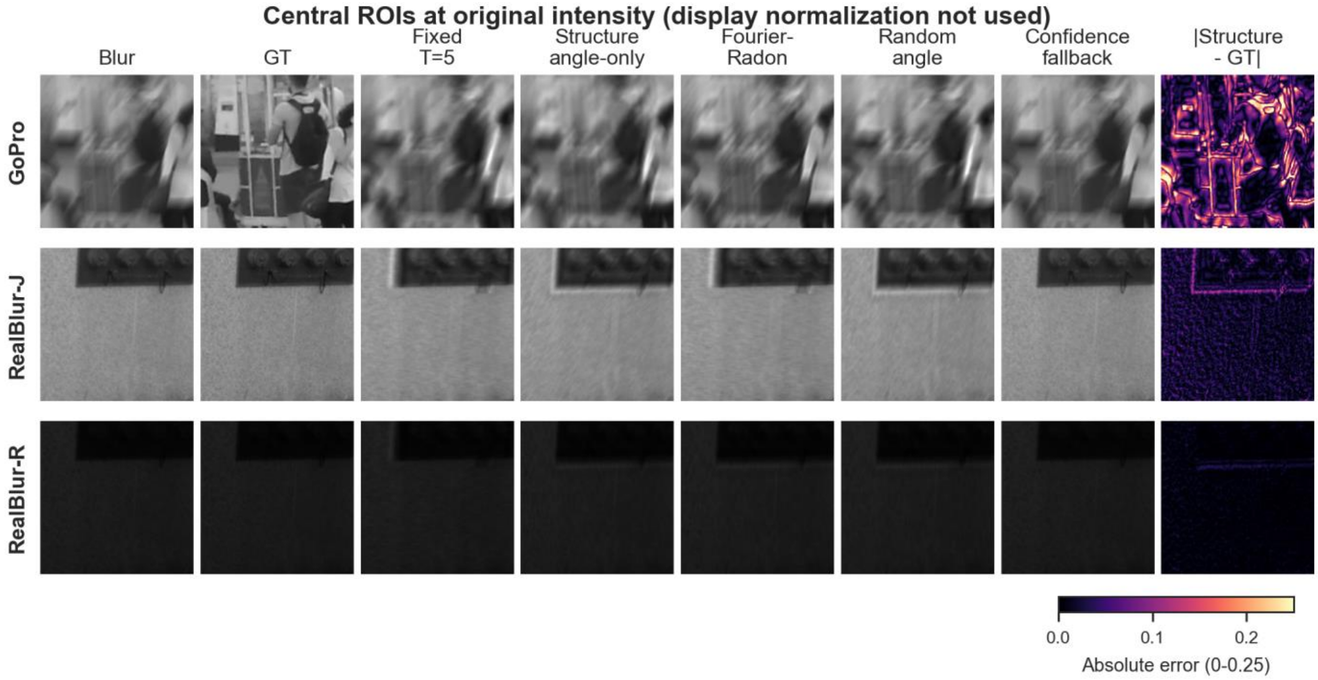

Figure S1. Enlarged central regions of interest for GoPro, RealBlur-J, and RealBlur-R. Panels retain original intensity; display-only normalization was not used for these panels or any metric. Method definitions and error scaling match main-text Figure 6. Image samples were adapted from the GoPro dataset (Nah et al., CC BY 4.0) and the RealBlur dataset (Rim et al., CC BY 4.0); cropping and panel assembly were produced by the authors.

Supplementary Figure S2

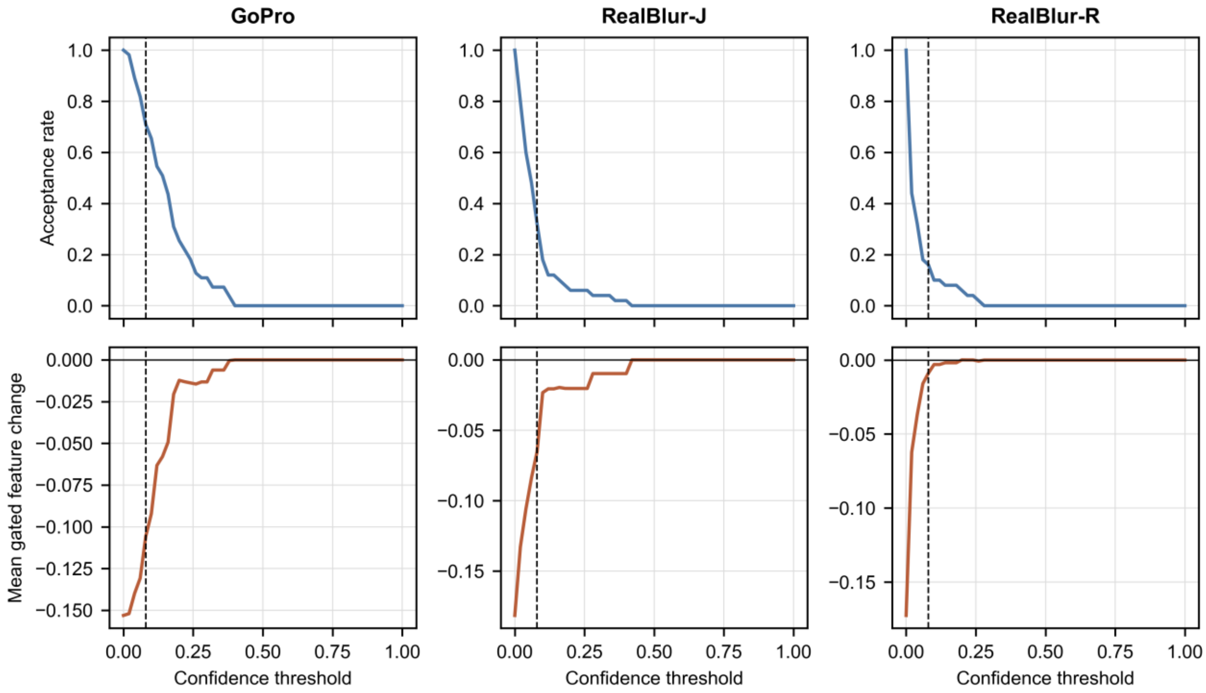

Figure S2. Feature-confidence threshold diagnostics on the previously fixed 155-image subset. The upper row shows acceptance rate and the lower row shows the mean gated change in the primary normalized ORB-homography endpoint. The dashed line is the retained c=0.08 threshold. Curves are descriptive sensitivity analyses and were not used to choose an optimal threshold.

Supplementary Figure S3

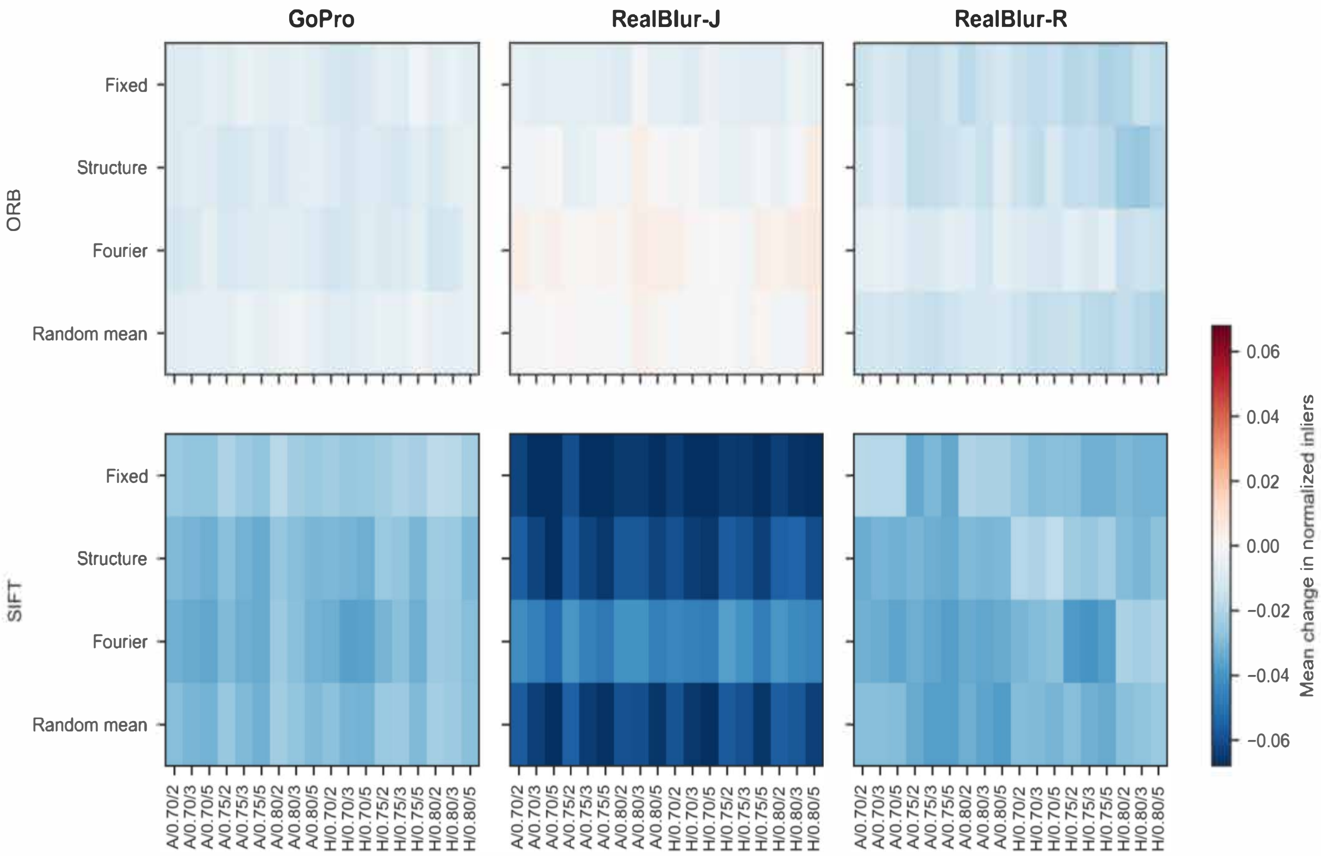

Figure S3. Correlated L = 5 feature-parameter sensitivity conditions. Rows are methods and columns combine geometry model (H, homography; A, affine), ratio threshold (r), and RANSAC threshold in pixels (t). Color encodes the mean normalized-inlier change. The 18 columns share images and overlapping settings and are correlated sensitivity conditions rather than independent replications. This heatmap was moved from the main text to keep the primary argument focused on direction, kernel length, raw/normalized evidence, confidence, and failure states.

Supplementary Material S1

Feature-Level Reliability of Directional-Kernel Richardson-Lucy Deblurring under Kernel-Length and Direction Controls

Xiangchen Ku, Runqing Xue, and Yichen Liang
Journal: Sensors; Section: Sensing and Imaging

File and Table Index

Figures S1-S3 appear before this index. Complete editable supplementary tables, including the lettered subtables listed below, and their source-level worksheets are supplied in Supplementary_Tables_S1-S38.xlsx.

Element	Description
Table S1	All-pair grayscale method summary.
Table S2	Ten-seed random-angle summary.
Table S3	Multi-seed LPIPS direct contrasts.
Table S4	Confidence discrimination and bootstrap diagnostics.
Table S5	Full-size boundary sensitivity.
Table S6	Synthetic axial direction calibration.
Table S7	Direct direction-source contrasts.
Table S8	Oracle-angle upper-bound summary.
Table S9	Kernel-length sensitivity.
Table S10	Wiener balance sensitivity.
Table S11	Full-size RGB sensitivity.
Table S12	GoPro sequence and leave-one-sequence-out robustness.
Table S13	Full ORB/SIFT baseline feature decomposition.
Table S14	Geometry success, failure counts, valid samples, and failure reasons.
Tables S15a-S15b	Detector, ratio-test, RANSAC, geometry-model, and edge-tolerance sensitivity.
Tables S16a-S16b	Confidence acceptance/rejection and feature/PSNR ranking diagnostics.
Tables S17a-S17b	GoPro sequence means, exact sign tests, and leave-one-sequence-out ranges.
Table S18	Direct L = 5 versus L = 13 paired cluster comparisons.
Tables S19a-S19b	L = 5 random-seed summaries and estimated-minus-random contrasts.
Tables S20a-S20b	Blur Input-success-only sensitivity and geometry-transition matrices.

Supplementary Material S1

Feature-Level Reliability of Directional-Kernel Richardson-Lucy Deblurring under Kernel-Length and Direction Controls

Xiangchen Ku, Runqing Xue, and Yichen Liang
Journal: Sensors; Section: Sensing and Imaging

File and Table Index (continued)

Element	Description
Tables S21a-S21b	Baseline, candidate, raw, normalized, relative, and success-rate decomposition.
Tables S22a-S22b	Correlated parameter-condition effects and factor-range summaries.
Tables S23a-S23b	Cluster-aware feature-confidence AUC/AP intervals and threshold diagnostics.
Tables S24a-S24j	Complete all-pair distributions and secondary restoration controls.
Table S25	Unique-image random-transition summary.
Table S26	ORB nfeatures = 2000 cap-saturation audit.
Table S27	Fixed-subset group coverage and Blur Input distribution audit.
Table S28	ORB nfeatures = 1000/2000/4000 sensitivity.
Table S29	Official NAFNet pixel and feature controls.
Table S30	Exact GoPro sequence-level randomization over all 2^11 assignments.
Table S31	Extended ORB cap sequence through nfeatures = 24000 and stopping audit.
Table S32	Official Restormer pixel and feature controls.
Table S33	Ground-truth-assisted joint angle-length oracle contrasts.
Table S34	RL discrepancy-stopping and RL-TV calibration/validation results.
Table S35	Ground-truth task-level RGB-D odometry records and inference.
Table S36	Subset coverage, analysis hierarchy, and BH-FDR-adjusted contrasts.
Tables S37a-S37c	GT-referenced distortion metrics, group summaries, and feature associations.
Tables S38a-S38c	Group effects and precision/MDE summaries for primary and secondary feature contrasts.

Supplementary Material S1

Feature-Level Reliability of Directional-Kernel Richardson-Lucy Deblurring under Kernel-Length and Direction Controls

Xiangchen Ku, Runqing Xue, and Yichen Liang
Journal: Sensors; Section: Sensing and Imaging

Supplementary Data and Code S1

Supplementary_Data_and_Code_S1.zip contains scripts, processed CSV records, integrity tests, environment records, sanitized manifests, figure source data, task-level RGB-D records, and rerun instructions. The integrated archive now also contains the v13 distortion, feature-association, and precision/MDE records plus their validation scripts.

Third-party public images, restored frames, repositories, and checkpoint weights are not redistributed. The distortion analysis is post hoc and supports association rather than causal mediation. The MDE is a precision descriptor, not an equivalence margin. Random seeds are averaged within image or sequence before group-level inference.

Supplementary Table S35: Ground-Truth Task-Level RGB-D Odometry

Article: Feature-Level Reliability of Directional-Kernel Richardson–Lucy Deblurring under Kernel-Length and Direction Controls

Journal: Sensors; **Section:** Sensing and Imaging; **Authors:** Xiangchen Ku, Runqing Xue, and Yichen Liang

Ten frozen TUM RGB-D sequences were evaluated with trajectory-conditioned synthetic 20-ms exposures. The sequence was the inferential unit. Random-angle seeds were averaged within sequence before inference. Negative Delta ATE favors preprocessing. Full endpoint records, wild-cluster intervals, exact sign tests, leave-one-out ranges, protocol details, and Figure 8 source data are provided in the editable workbook and data/code archive.

Detector	Branch	Blur ATE	Processed ATE	Delta ATE [95% CI]	Improved sequences	Exact sign-flip p
ORB	Fixed RL L=5	0.184	0.203	+0.020 [+0.007, +0.039]	1/10	0.0039
ORB	Structure tensor L=5	0.184	0.188	+0.004 [-0.006, +0.015]	3/10	0.5371
ORB	Fourier-Radon L=5	0.184	0.188	+0.005 [-0.008, +0.018]	5/10	0.5000
ORB	Fixed RL L=13	0.184	0.234	+0.051 [+0.010, +0.101]	2/10	0.0352
ORB	Structure tensor L=13	0.184	0.224	+0.040 [+0.013, +0.071]	2/10	0.0254
ORB	Confidence fallback	0.184	0.224	+0.041 [+0.017, +0.070]	0/10	0.0020
ORB	Random angle L=5 (10-seed mean)	0.184	0.196	+0.012 [+0.004, +0.020]	2/10	0.0254
ORB	NAFNet	0.184	0.189	+0.006 [-0.010, +0.022]	5/10	0.5371
ORB	Restormer	0.184	0.192	+0.009 [-0.013, +0.043]	5/10	0.9375
SIFT	Fixed RL L=5	0.152	0.160	+0.008 [+0.001, +0.017]	4/10	0.0605
SIFT	Structure tensor L=5	0.152	0.160	+0.008 [-0.004, +0.020]	4/10	0.2422
SIFT	Fourier-Radon L=5	0.152	0.174	+0.022 [-0.003, +0.053]	3/10	0.2207
SIFT	Fixed RL L=13	0.152	0.182	+0.030 [+0.000, +0.068]	3/10	0.1602
SIFT	Structure tensor L=13	0.152	0.181	+0.029 [+0.003, +0.053]	2/10	0.0527
SIFT	Confidence fallback	0.152	0.188	+0.036 [+0.015, +0.060]	2/10	0.0078
SIFT	Random angle L=5 (10-seed mean)	0.152	0.159	+0.007 [-0.001, +0.015]	3/10	0.1523
SIFT	NAFNet	0.152	0.144	-0.008 [-0.025, +0.008]	7/10	0.4199
SIFT	Restormer	0.152	0.145	-0.008 [-0.022, +0.003]	5/10	0.3066

Supplementary Table S36

Fixed-subset coverage, analysis hierarchy, and the revision-defined BH-FDR family. Complete editable values are provided in Supplementary_Tables_S1-S36.xlsx.

Dataset	Full n	Subset n	Full groups	Subset groups	ΔPSNR	ΔSSIM
GoPro	1111	55	11	11	0.0551	-0.0046
RealBlur_J	980	50	50	50	-0.2473	-0.0161
RealBlur_R	980	50	50	50	-0.1196	-0.0008

BH-FDR result: 0 of 12 secondary contrasts met $q < 0.05$. The minimum adjusted q was 0.109.

Supplementary Material S1

Feature-Level Reliability of Directional-Kernel Richardson-Lucy Deblurring under Kernel-Length and Direction Controls

Xiangchen Ku, Runqing Xue, and Yichen Liang
Journal: Sensors; Section: Sensing and Imaging

Supplementary Table S37b: GT-Referenced Distortion Group Summary

Values are sequence/scene-group means [two-sided group-level 95% CI] relative to Blur Input. Positive values indicate increased distortion. rho is the image-level Spearman association with the normalized ORB-homography inlier change, with cluster-bootstrap 95% CI. Complete per-image values, all L = 13 controls, and all associations are in the editable workbook.

Dataset	Method	Edge envelope	Gradient distortion	Laplacian residual	rho(gradient, Delta q)
GoPro	Fixed L5	0.0008 [0.0001, 0.0015]	0.0062 [-0.0179, 0.0303]	0.0478 [0.0134, 0.0821]	-0.415 [-0.627, -0.170]
GoPro	Structure L5	0.0010 [0.0001, 0.0019]	0.0075 [-0.0198, 0.0348]	0.0483 [0.0172, 0.0795]	-0.530 [-0.707, -0.263]
GoPro	FourierRadon L5	0.0010 [0.0003, 0.0018]	0.0118 [-0.0152, 0.0388]	0.0604 [0.0231, 0.0976]	-0.565 [-0.753, -0.274]
RealBlur-J	Fixed L5	0.0017 [0.0011, 0.0023]	0.0169 [0.0013, 0.0324]	0.0628 [0.0390, 0.0867]	-0.532 [-0.734, -0.247]
RealBlur-J	Structure L5	0.0013 [0.0009, 0.0016]	0.0120 [-0.0007, 0.0248]	0.0589 [0.0400, 0.0778]	-0.310 [-0.591, 0.022]
RealBlur-J	FourierRadon L5	0.0013 [0.0007, 0.0019]	0.0113 [-0.0023, 0.0250]	0.0574 [0.0362, 0.0787]	-0.670 [-0.829, -0.436]
RealBlur-R	Fixed L5	0.0005 [0.0003, 0.0006]	0.0202 [0.0086, 0.0318]	0.0771 [0.0595, 0.0947]	-0.222 [-0.507, 0.081]
RealBlur-R	Structure L5	0.0006 [0.0004, 0.0008]	0.0324 [0.0197, 0.0451]	0.1139 [0.0967, 0.1310]	-0.085 [-0.392, 0.232]
RealBlur-R	FourierRadon L5	0.0004 [0.0003, 0.0006]	0.0231 [0.0111, 0.0350]	0.0953 [0.0767, 0.1138]	-0.297 [-0.552, 0.015]

Supplementary Material S1

Feature-Level Reliability of Directional-Kernel Richardson-Lucy Deblurring under Kernel-Length and Direction Controls

Xiangchen Ku, Runqing Xue, and Yichen Liang
Journal: Sensors; Section: Sensing and Imaging

Supplementary Table S38b: Primary L = 5 Contrasts versus Blur Input

Effects are group means. The MDE uses a two-sided alpha = 0.05 test, 80% power, and the observed between-group standard deviation. It describes attainable precision and is not an equivalence margin.

Dataset	Detector	Method	Contrast	n groups	Mean effect [95% CI]	CI half-width	80% MDE	MDE / reference
GoPro	ORB	Fixed L5	Fixed L5 minus Blur Input	11	-0.0082 [-0.0219, 0.0055]	0.0137	0.0191	7.5%
GoPro	ORB	FourierRadon L5	FourierRadon L5 minus Blur Input	11	-0.0091 [-0.0230, 0.0048]	0.0139	0.0194	7.6%
GoPro	ORB	Structure L5	Structure L5 minus Blur Input	11	-0.0121 [-0.0248, 0.0007]	0.0128	0.0178	7.0%
RealBlur-J	ORB	Fixed L5	Fixed L5 minus Blur Input	50	-0.0071 [-0.0190, 0.0048]	0.0119	0.0169	6.0%
RealBlur-J	ORB	FourierRadon L5	FourierRadon L5 minus Blur Input	50	-0.0017 [-0.0138, 0.0105]	0.0122	0.0173	6.2%
RealBlur-J	ORB	Structure L5	Structure L5 minus Blur Input	50	-0.0047 [-0.0156, 0.0062]	0.0109	0.0156	5.5%
RealBlur-R	ORB	Fixed L5	Fixed L5 minus Blur Input	50	-0.0180 [-0.0342, -0.0018]	0.0162	0.0230	8.1%
RealBlur-R	ORB	FourierRadon L5	FourierRadon L5 minus Blur Input	50	-0.0100 [-0.0245, 0.0045]	0.0145	0.0206	7.2%
RealBlur-R	ORB	Structure L5	Structure L5 minus Blur Input	50	-0.0156 [-0.0278, -0.0033]	0.0123	0.0175	6.1%

Supplementary Material S1

Feature-Level Reliability of Directional-Kernel Richardson-Lucy Deblurring under Kernel-Length and Direction Controls

Xiangchen Ku, Runqing Xue, and Yichen Liang

Journal: Sensors; Section: Sensing and Imaging

Supplementary Table S38c: Secondary Estimated-Direction Contrasts versus 10-Seed Random Mean

Effects are group means. The MDE uses a two-sided alpha = 0.05 test, 80% power, and the observed between-group standard deviation. It describes attainable precision and is not an equivalence margin.

Dataset	Detector	Method	Contrast	n groups	Mean effect [95% CI]	CI half-width	80% MDE	MDE / reference
GoPro	ORB	FourierRadon L5	FourierRadon L5 minus 10-seed random mean	11	-0.0038 [-0.0105, 0.0030]	0.0068	0.0094	3.8%
GoPro	ORB	Structure L5	Structure L5 minus 10-seed random mean	11	-0.0067 [-0.0128, -0.0007]	0.0060	0.0084	3.4%
GoPro	SIFT	FourierRadon L5	FourierRadon L5 minus 10-seed random mean	11	-0.0035 [-0.0145, 0.0076]	0.0110	0.0154	8.0%
GoPro	SIFT	Structure L5	Structure L5 minus 10-seed random mean	11	-0.0022 [-0.0136, 0.0092]	0.0114	0.0159	8.3%
RealBlur-J	ORB	FourierRadon L5	FourierRadon L5 minus 10-seed random mean	50	0.0008 [-0.0065, 0.0081]	0.0073	0.0104	3.7%
RealBlur-J	ORB	Structure L5	Structure L5 minus 10-seed random mean	50	-0.0022 [-0.0103, 0.0059]	0.0081	0.0115	4.1%
RealBlur-J	SIFT	FourierRadon L5	FourierRadon L5 minus 10-seed random mean	50	0.0188 [0.0048, 0.0328]	0.0140	0.0200	8.9%
RealBlur-J	SIFT	Structure L5	Structure L5 minus 10-seed random mean	50	0.0008 [-0.0112, 0.0129]	0.0121	0.0171	7.6%
RealBlur-R	ORB	FourierRadon L5	FourierRadon L5 minus 10-seed random mean	50	0.0077 [-0.0025, 0.0179]	0.0102	0.0145	5.4%
RealBlur-R	ORB	Structure L5	Structure L5 minus 10-seed random mean	50	0.0021 [-0.0051, 0.0094]	0.0073	0.0104	3.9%
RealBlur-R	SIFT	FourierRadon L5	FourierRadon L5 minus 10-seed random mean	50	-0.0073 [-0.0316, 0.0170]	0.0243	0.0346	16.9%
RealBlur-R	SIFT	Structure L5	Structure L5 minus 10-seed random mean	50	0.0071 [-0.0069, 0.0210]	0.0140	0.0199	9.7%
