# Supplementary material for: Feature-Level Reliability of Directional-Kernel Richardson–Lucy Deblurring Under Kernel-Length and Direction Controls
Source: Sensors (Basel). 2026 Aug 19;26(16):5249. doi: 10.3390/s26165249 (PMC13517317; doi:10.3390/s26165249)
Supplement: Supplementary file 1 [file sensors-26-05249-s001.zip › Supplementary_Data_and_Code_S1.zip!/figure_source_data/Figure_7_feature_robustness_L5_L13.pdf]

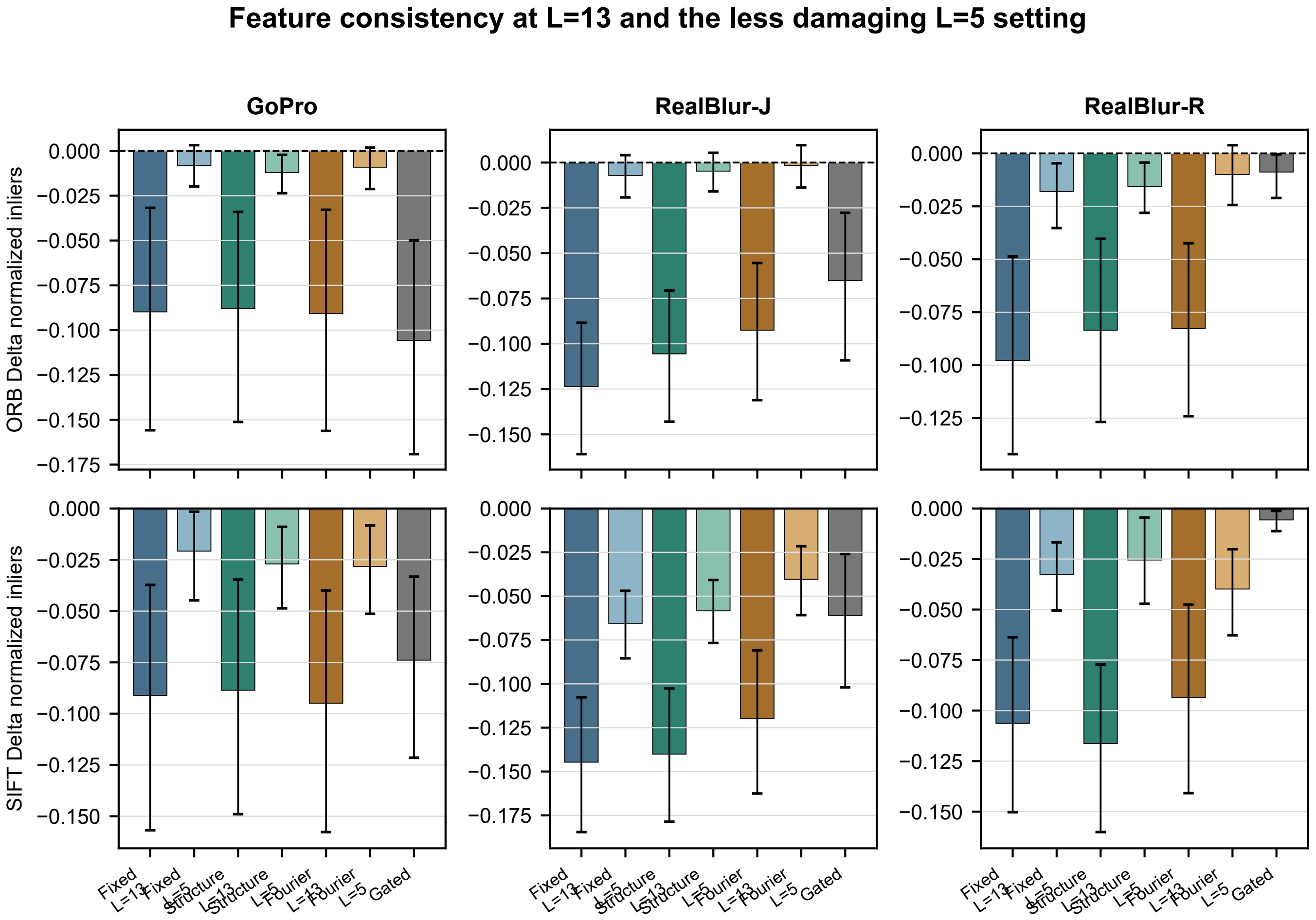

Feature consistency at L=13 and the less damaging L=5 setting
GoPro
RealBlur-J
RealBlur-R
ORB Delta normalized inliers
SIFT Delta normalized inliers
Fixed L=13
Fixed L=5
Structure L=13
Structure L=5
Fourier L=13
Fourier L=5
Gated
