# Supplementary material for: Feature-Level Reliability of Directional-Kernel Richardson–Lucy Deblurring Under Kernel-Length and Direction Controls
Source: Sensors (Basel). 2026 Aug 19;26(16):5249. doi: 10.3390/s26165249 (PMC13517317; doi:10.3390/s26165249)
Supplement: Supplementary file 1 [file sensors-26-05249-s001.zip › Supplementary_Data_and_Code_S1.zip!/figure_source_data/Figure_7_feature_robustness_random_L5.pdf]

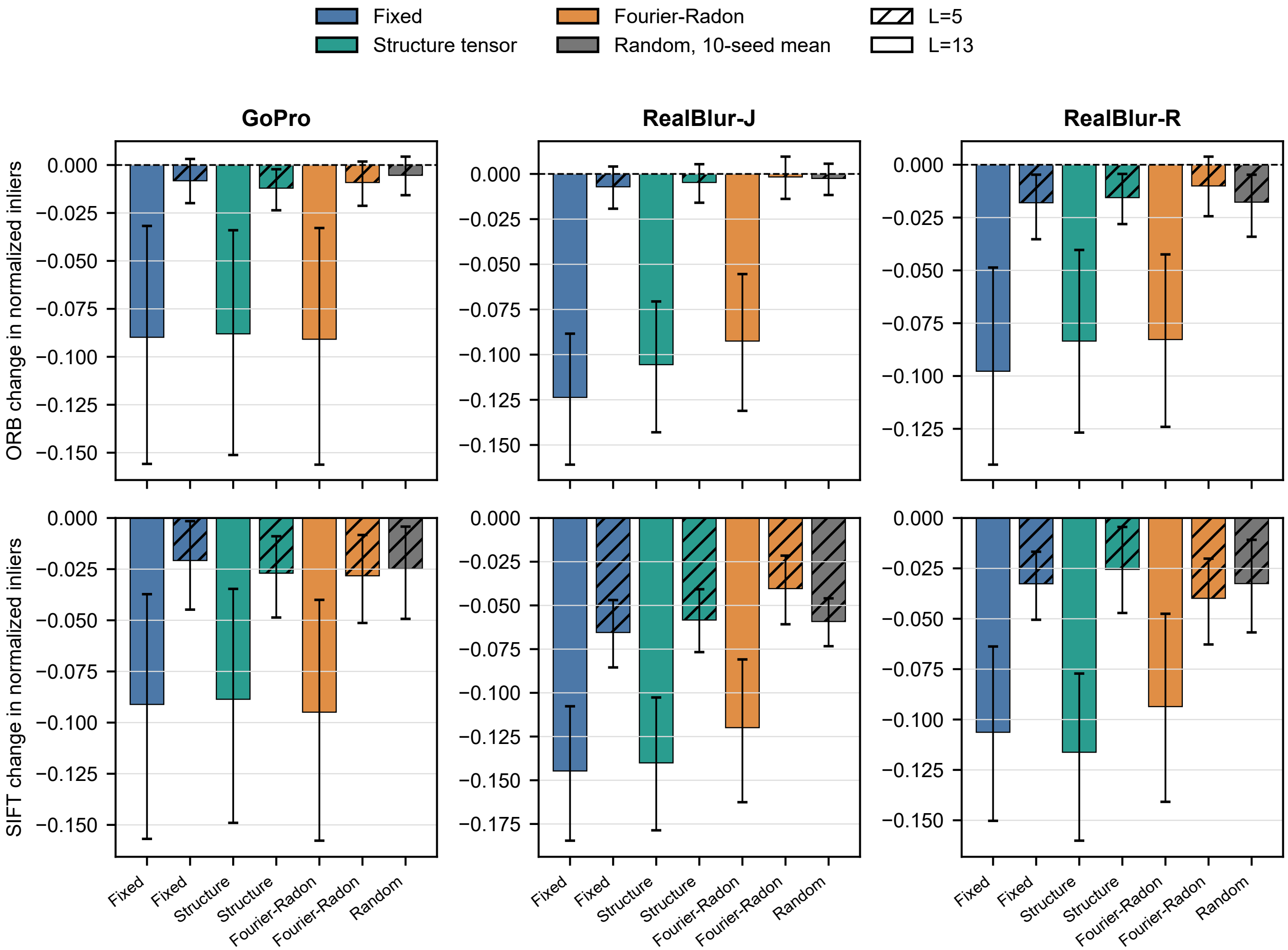
Fixed
Structure tensor
Fourier-Radon
Random, 10-seed mean
L=5
L=13
GoPro
RealBlur-J
RealBlur-R
ORB change in normalized inliers
SIFT change in normalized inliers
Fixed
Fixed
Structure
Structure
Fourier-Radon
Fourier-Radon
Random
