# Supplementary material for: Feature-Level Reliability of Directional-Kernel Richardson–Lucy Deblurring Under Kernel-Length and Direction Controls
Source: Sensors (Basel). 2026 Aug 19;26(16):5249. doi: 10.3390/s26165249 (PMC13517317; doi:10.3390/s26165249)
Supplement: Supplementary file 1 [file sensors-26-05249-s001.zip › Supplementary_Data_and_Code_S1.zip!/figure_source_data/Figure_8_feature_parameter_heatmap.pdf]

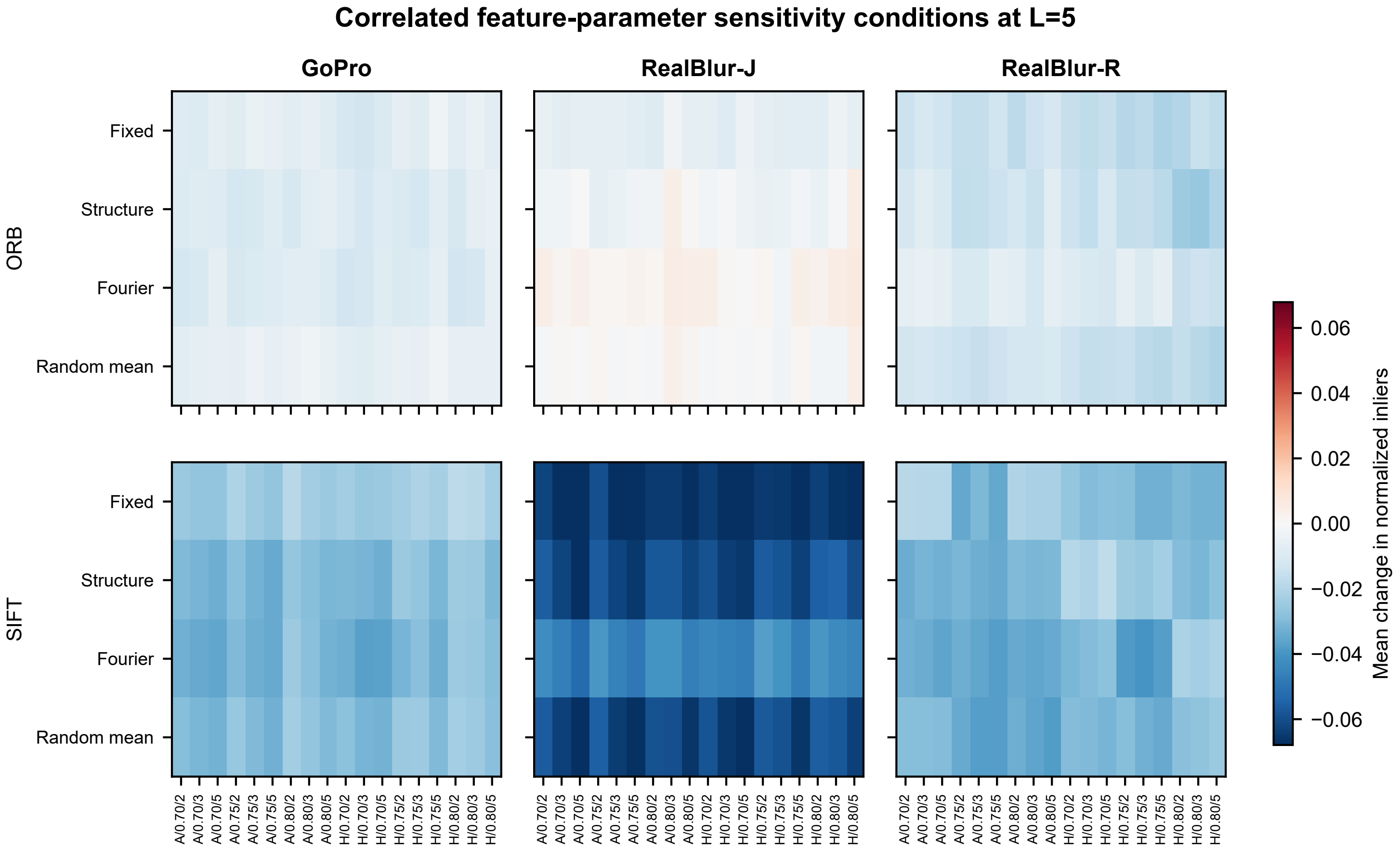
Correlated feature-parameter sensitivity conditions at L=5
GoPro
RealBlur-J
RealBlur-R
ORB
SIFT
Fixed
Structure
Fourier
Random mean
A/0.70/2
A/0.70/3
A/0.70/5
A/0.75/2
A/0.75/3
A/0.75/5
A/0.80/2
A/0.80/3
A/0.80/5
H/0.70/2
H/0.70/3
H/0.70/5
H/0.75/2
H/0.75/3
H/0.75/5
H/0.80/2
H/0.80/3
H/0.80/5
Mean change in normalized inliers
0.06
0.04
0.02
0.00
−0.02
−0.04
−0.06
