# Supplementary material for: Feature-Level Reliability of Directional-Kernel Richardson–Lucy Deblurring Under Kernel-Length and Direction Controls
Source: Sensors (Basel). 2026 Aug 19;26(16):5249. doi: 10.3390/s26165249 (PMC13517317; doi:10.3390/s26165249)
Supplement: Supplementary file 1 [file sensors-26-05249-s001.zip › Supplementary_Data_and_Code_S1.zip!/figure_source_data/Supplementary_Figure_S2_confidence_feature_thresholds.pdf]

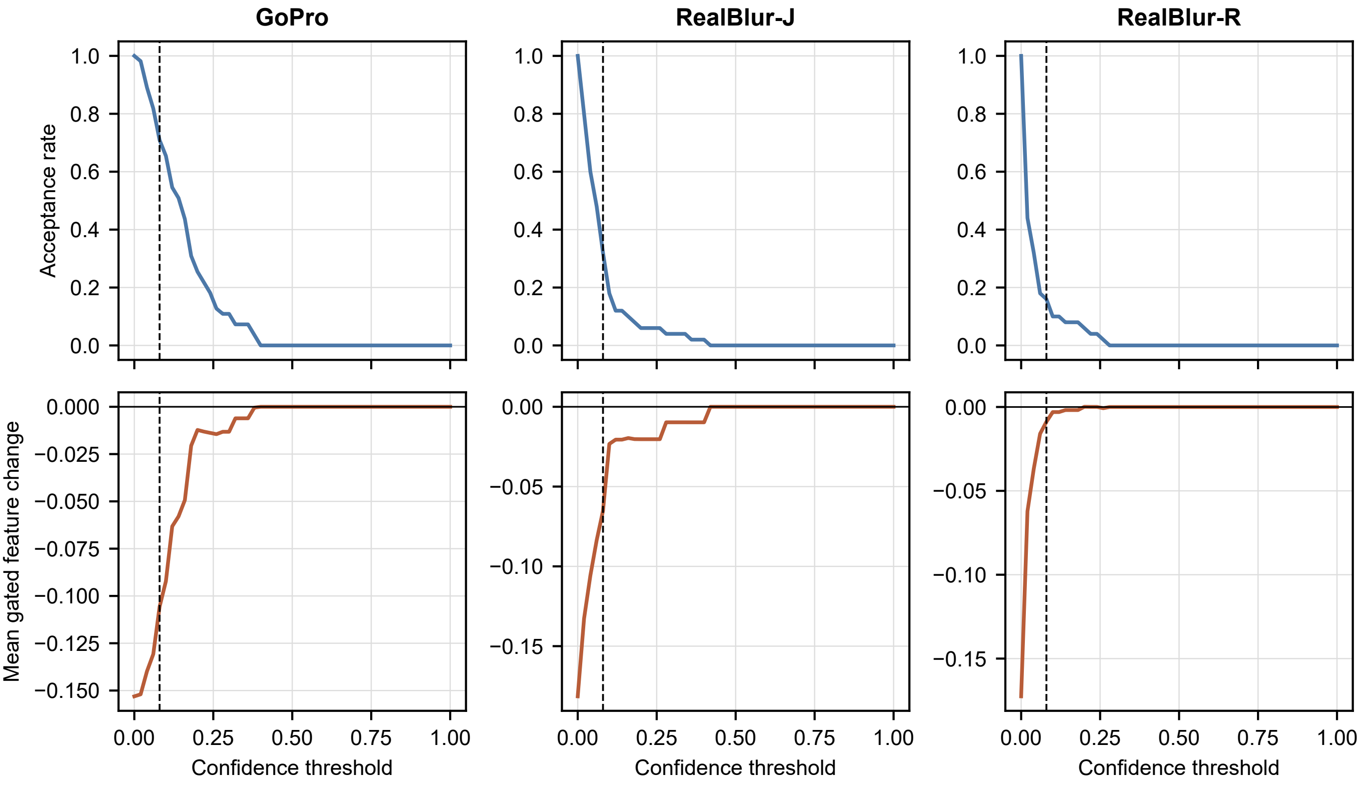
GoPro
RealBlur-J
RealBlur-R
Acceptance rate
Mean gated feature change
Confidence threshold
1.0
0.8
0.6
0.4
0.2
0.0
0.000
−0.025
−0.050
−0.075
−0.100
−0.125
−0.150
0.00
−0.05
−0.10
−0.15
0.00
0.25
0.50
0.75
1.00
