# Supplementary material for: Feature-Level Reliability of Directional-Kernel Richardson–Lucy Deblurring Under Kernel-Length and Direction Controls
Source: Sensors (Basel). 2026 Aug 19;26(16):5249. doi: 10.3390/s26165249 (PMC13517317; doi:10.3390/s26165249)
Supplement: Supplementary file 1 [file sensors-26-05249-s001.zip › Supplementary_Data_and_Code_S1.zip!/figure_source_data/camera_feature_reliability/Figure_1_camera_sensing_workflow.pdf]

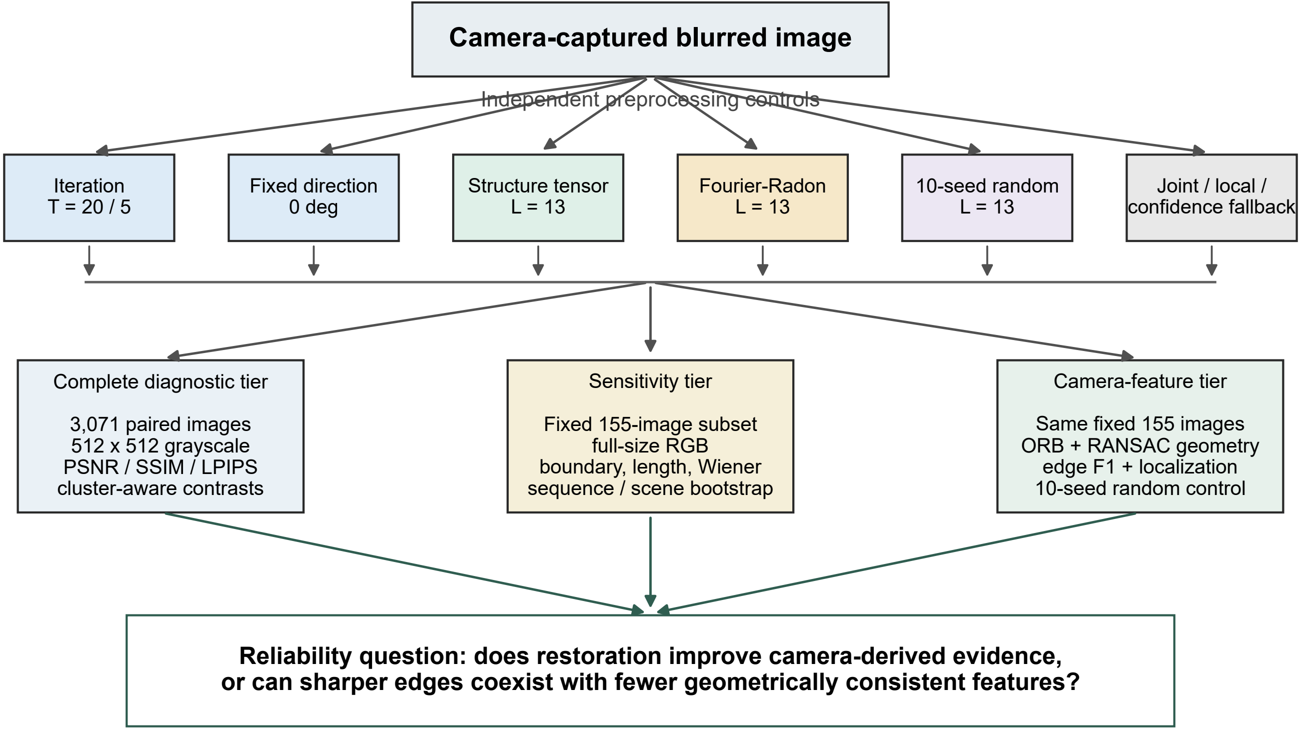
Camera-captured blurred image
Independent preprocessing controls
Iteration
T = 20 / 5
Fixed direction
0 deg
Structure tensor
L = 13
Fourier-Radon
L = 13
10-seed random
L = 13
Joint / local /
confidence fallback
Complete diagnostic tier
3,071 paired images
512 x 512 grayscale
PSNR / SSIM / LPIPS
cluster-aware contrasts
Sensitivity tier
Fixed 155-image subset
full-size RGB
boundary, length, Wiener
sequence / scene bootstrap
Camera-feature tier
Same fixed 155 images
ORB + RANSAC geometry
edge F1 + localization
10-seed random control
Reliability question: does restoration improve camera-derived evidence,
or can sharper edges coexist with fewer geometrically consistent features?
