# Supplementary material for: Feature-Level Reliability of Directional-Kernel Richardson–Lucy Deblurring Under Kernel-Length and Direction Controls
Source: Sensors (Basel). 2026 Aug 19;26(16):5249. doi: 10.3390/s26165249 (PMC13517317; doi:10.3390/s26165249)

Camera-feature changes relative to Blur Input (fixed 155-image subset)

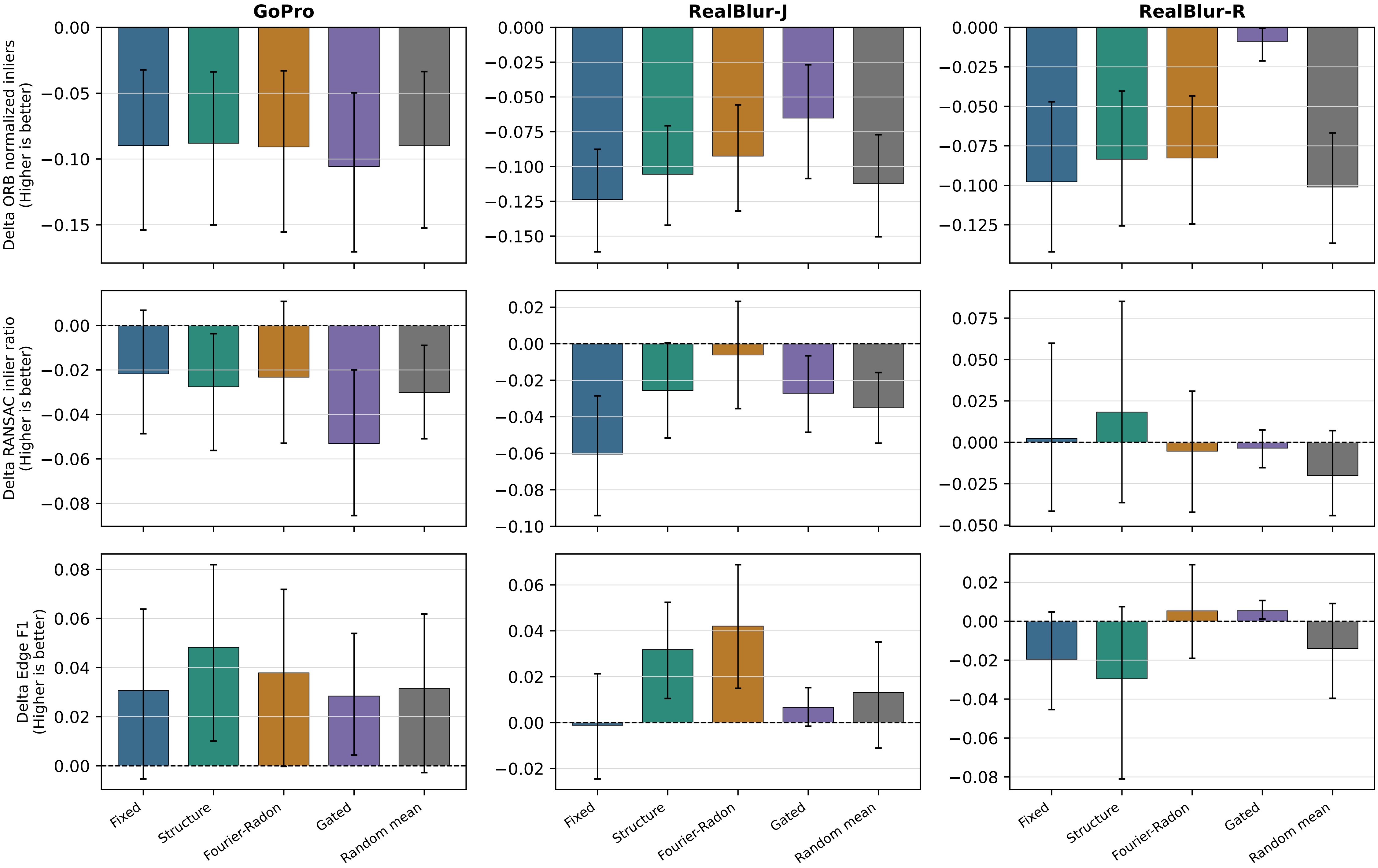

Supplement: Supplementary file 1 [file sensors-26-05249-s001.zip › Supplementary_Data_and_Code_S1.zip!/figure_source_data/camera_feature_reliability/Figure_7_camera_feature_reliability.pdf]
