# Supplementary material for: Feature-Level Reliability of Directional-Kernel Richardson–Lucy Deblurring Under Kernel-Length and Direction Controls
Source: Sensors (Basel). 2026 Aug 19;26(16):5249. doi: 10.3390/s26165249 (PMC13517317; doi:10.3390/s26165249)
Supplement: Supplementary file 1 [file sensors-26-05249-s001.zip › Supplementary_Data_and_Code_S1.zip!/tum_rgbd_task_s35/results/tum_rgbd_task_primary_ate_effects.pdf]

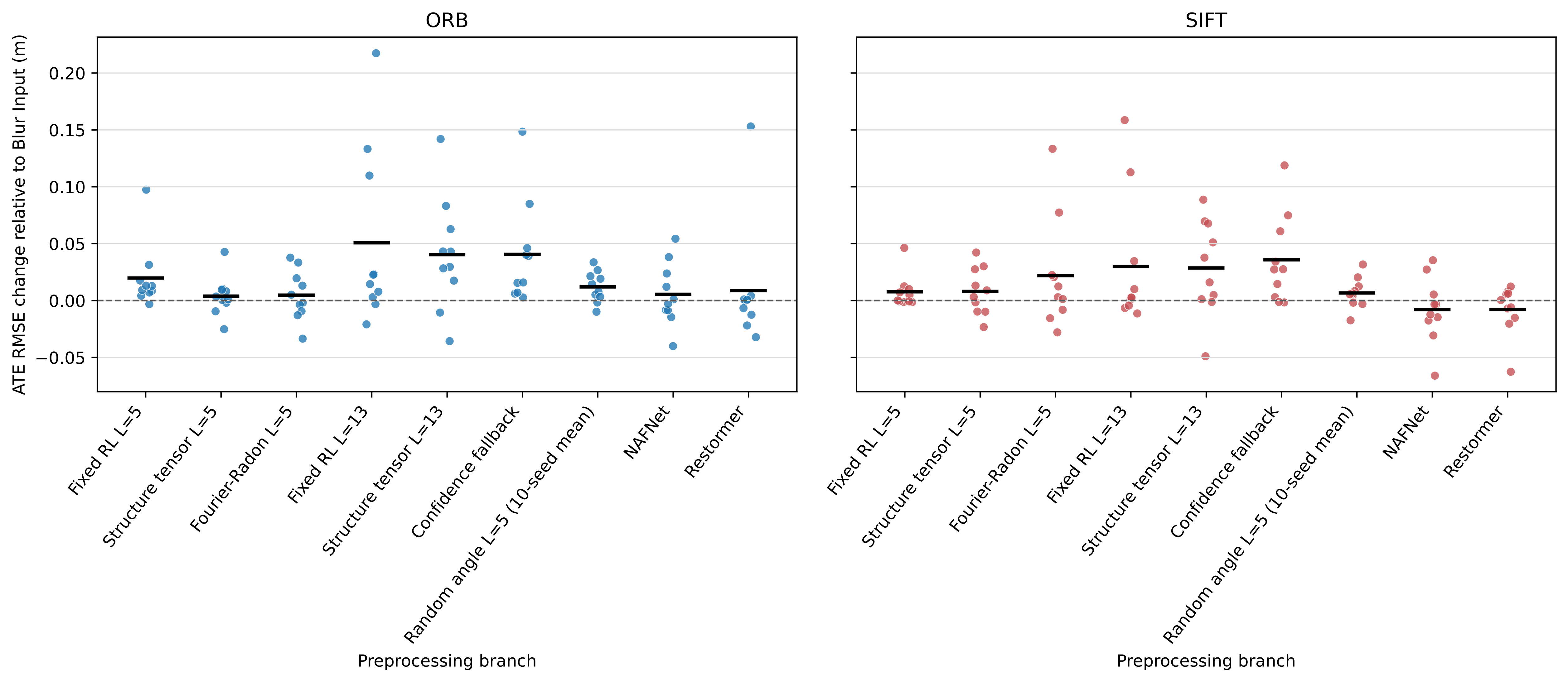
ORB
SIFT
ATE RMSE change relative to Blur Input (m)
0.20
0.15
0.10
0.05
0.00
−0.05
Fixed RL L=5
Structure tensor L=5
Fourier-Radon L=5
Fixed RL L=13
Structure tensor L=13
Confidence fallback
Random angle L=5 (10-seed mean)
NAFNet
Restormer
Preprocessing branch
